# A review of brain imaging biomarker genomics in Alzheimer’s disease: implementation and perspectives

**DOI:** 10.1186/s40035-022-00315-z

**Published:** 2022-09-15

**Authors:** Lanlan Li, Xianfeng Yu, Can Sheng, Xueyan Jiang, Qi Zhang, Ying Han, Jiehui Jiang

**Affiliations:** 1 School of Information and Communication Engineering, Shanghai University, Shanghai, 200444 China; 2 School of Biomedical Engineering, Hainan University, Haikou, 570228 China; 3 Department of Neurology, Xuanwu Hospital of Capital Medical University, Beijing, 100053 China; 4 Institute of Biomedical Engineering, School of Life Science, Shanghai University, Shanghai, 200444 China

**Keywords:** Imaging biomarker genomics, Alzheimer’s disease, Evolving technologies, Implementation

## Abstract

Alzheimer’s disease (AD) is a progressive neurodegenerative disease with phenotypic changes closely associated with both genetic variants and imaging pathology. Brain imaging biomarker genomics has been developed in recent years to reveal potential AD pathological mechanisms and provide early diagnoses. This technique integrates multimodal imaging phenotypes with genetic data in a noninvasive and high-throughput manner. In this review, we summarize the basic analytical framework of brain imaging biomarker genomics and elucidate two main implementation scenarios of this technique in AD studies: (1) exploring novel biomarkers and seeking mutual interpretability and (2) providing a diagnosis and prognosis for AD with combined use of machine learning methods and brain imaging biomarker genomics. Importantly, we highlight the necessity of brain imaging biomarker genomics, discuss the strengths and limitations of current methods, and propose directions for development of this research field.

**Supplementary Information:**

The online version contains supplementary material available at 10.1186/s40035-022-00315-z.

## Background

Alzheimer’s disease (AD), as the most common form of dementia, is an irreversible neurodegenerative disease. Epidemiological investigations have reported that about 55 million people worldwide live with AD and other types of dementia today [1]. The number is expected to reach 78 million by 2030 (World Alzheimer Report 2021, www.alz.co.uk). The primary clinical manifestations of AD include progressive impairments in memory and other cognitive functions, accompanied by several pathophysiological changes, such as amyloid deposition and neurofibrillary tangle formation. However, the aetiology and pathogenesis leading to heterogeneity in these manifestations among AD patients remain unclear. In addition, no effective therapeutic strategies are available for AD [2]. High-throughput imaging and genomics studies can provide valid information on AD pathology, and gain insights into the early detection and treatment of AD patients, and thus have attracted much attention recently.

Genomic studies have been developed over three decades [3–5]. In 1984, Glenner et al. [6] first isolated amyloid-β (Aβ) peptide from plaques in AD patients, and this peptide was shown to be generated from the amyloid precursor protein (APP) through its sequential cleavage by two enzymes: β-secretase and γ-secretase [3]. This finding was later confirmed by genetic mutations in *APP* in 1991 [7] and presenilins (*PSEN1* and *PSEN2*) in 1995 [8, 9]. The above genomic studies support an evident molecular mechanism underlying AD, resulting in the amyloid hypothesis. Additionally, the apolipoprotein E (*APOE*) ɛ4 allele has been reported to be associated with AD risk [10]. APOE can bind to Aβ, which influences the clearance of soluble Aβ and Aβ aggregation [11, 12], and regulates Aβ metabolism [13]. Notably, APOE ɛ4 binds more rapidly than APOE ɛ3, resulting in accelerated formation of fibrils [14]. Furthermore, with the development of high-throughput sequencing technology, genome-wide association studies (GWAS) have identified thousands of risk variants related to complex diseases and traits, including AD [15–34]. These studies have improved the understanding of genetic complexity and provided insights into the molecular pathways of AD pathogenesis. However, significant results are not only dependent on sufficiently large sample sizes but also require further analysis of gene-to-disease specificity.

Alternatively, neuroimaging technologies [35, 36] such as structural magnetic resonance imaging (sMRI), functional MRI (fMRI), diffusion tensor imaging (DTI), and positron emission tomography (PET), enable noninvasive detection of brain degeneration from the perspective of brain structure and function. SMRI can provide accurate in vivo quantification of specific regions with cortical and subcortical grey matter (GM) atrophy and white matter (WM) lesions associated with AD pathology, even at the mild cognitive impairment (MCI) stage [37, 38]. DTI is another MRI technique that is sensitive to translational motion of water molecules throughout the brain, providing quantification of WM tissue microstructure and visualization of WM tract abnormalities in AD patients. FMRI can measure brain activity by detecting associated changes in blood flow when no task is being performed, and task fMRI focuses on activity activation. Moreover, PET scans can demonstrate characteristic patterns of amyloid load, tau burden and glucose metabolism in AD patients by using specific molecular imaging tracers. The advanced imaging technologies have played important roles in quantitative assessment of biomarkers and understanding processes underlying AD. The National Institute on Aging−Alzheimer’s Association (NIA−AA) outlined in 2018 an unbiased descriptive AD biomarker classification scheme, called the ATN (amyloid, tau, neurodegeneration) diagnosis framework [39]. However, due to the complex heterogeneity of AD, the interactions among accessible, objective imaging markers and the complete pathological loop that is formed remain unknown. The emerging field of imaging biomarker genomics that combines multimodal imaging and high-throughput sequencing technologies, is committed to analysing associations between imaging phenotypes and genomics data and using imaging phenotypes as intermediate phenotypes between genetic variants and clinical diagnosis to investigate the pathogenesis of AD. Hence, the imaging biomarker genomics approach can overcome the shortcomings of separate genomics or imaging analysis, in that it can confirm gene-to-disease specificity, promote the biological interpretability of pathological biomarkers, and contribute to the diagnosis, treatment and prevention of AD with multiscale imaging and genetic features.

**Fig. 1:**
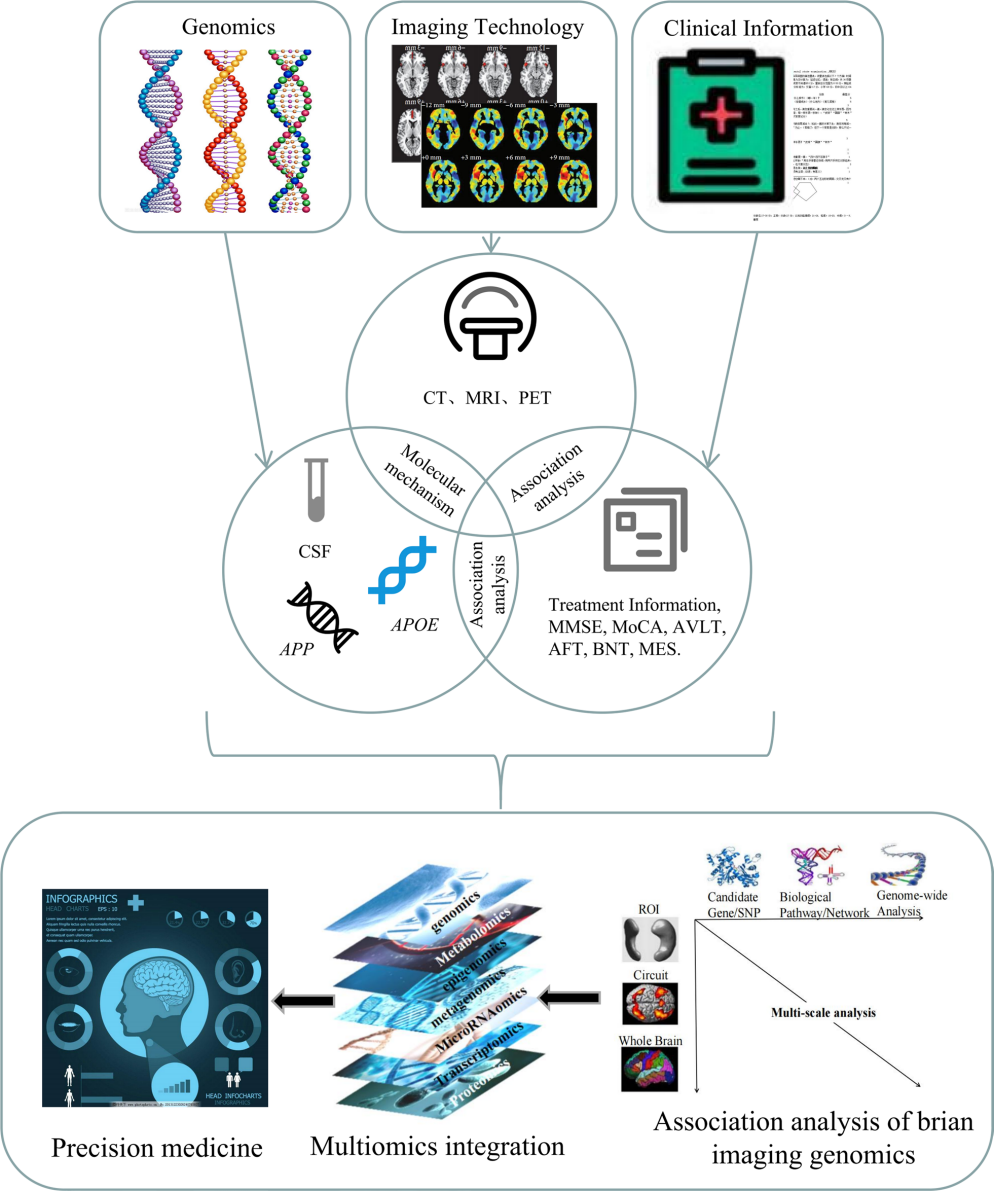
Landscape of advances of the AD imaging biomarker genomics field. This field covers genomics, imaging, and clinical information, ultimately pointing towards integrated diagnosis and precision medicine. *CSF* cerebrospinal fluid, *CT* computed tomography, *MMSE* mini-mental state examination, *MoCA* montreal cognitive assessment, *AVLT* auditory-verbal learning test, *AFT* animal fluency test, *BNT* boston naming test, *MES* memory and executive screening scale

When combined with clinical information, the imaging biomarker genomics approach may even facilitate precision medicine (Fig. 1). In this review, we provide a comprehensive summary of the brain imaging biomarker genomics approach, including (1) the basic analytical framework of brain imaging biomarker genomics studies and (2) implementation of this approach in AD based on the ATN framework, for exploring and validating AD biomarkers/variants and performing AD diagnosis and prognosis analysis. In particular, we introduce some key considerations relevant to studies using the brain imaging biomarker genomics approach and provide perspectives on the integration of neuroimaging and multiomics data and further methodological possibilities.

In particular, this study focuses on neuroimaging markers based on the ATN framework. Other biomarkers, such as various cerebrospinal fluid (CSF) biomarkers, electroencephalography (EEG) or magnetoencephalography (MEG) markers, are excluded. In addition, other risk factors for AD (e.g. sex, education, cognitive tests, etc.) will not be discussed in this paper.

## Methods

Literature was searched in Google Scholar and PubMed databases. Only human studies in English language, published from January 1991 (the publication year of earliest gene cloning of *APP* mutations) to December 2021 were reviewed. A total of 1095 records were yielded, of which 910 records were left after duplicate removal. A thorough description of the search strategy is provided in Additional file 1.

**Fig. 2:**
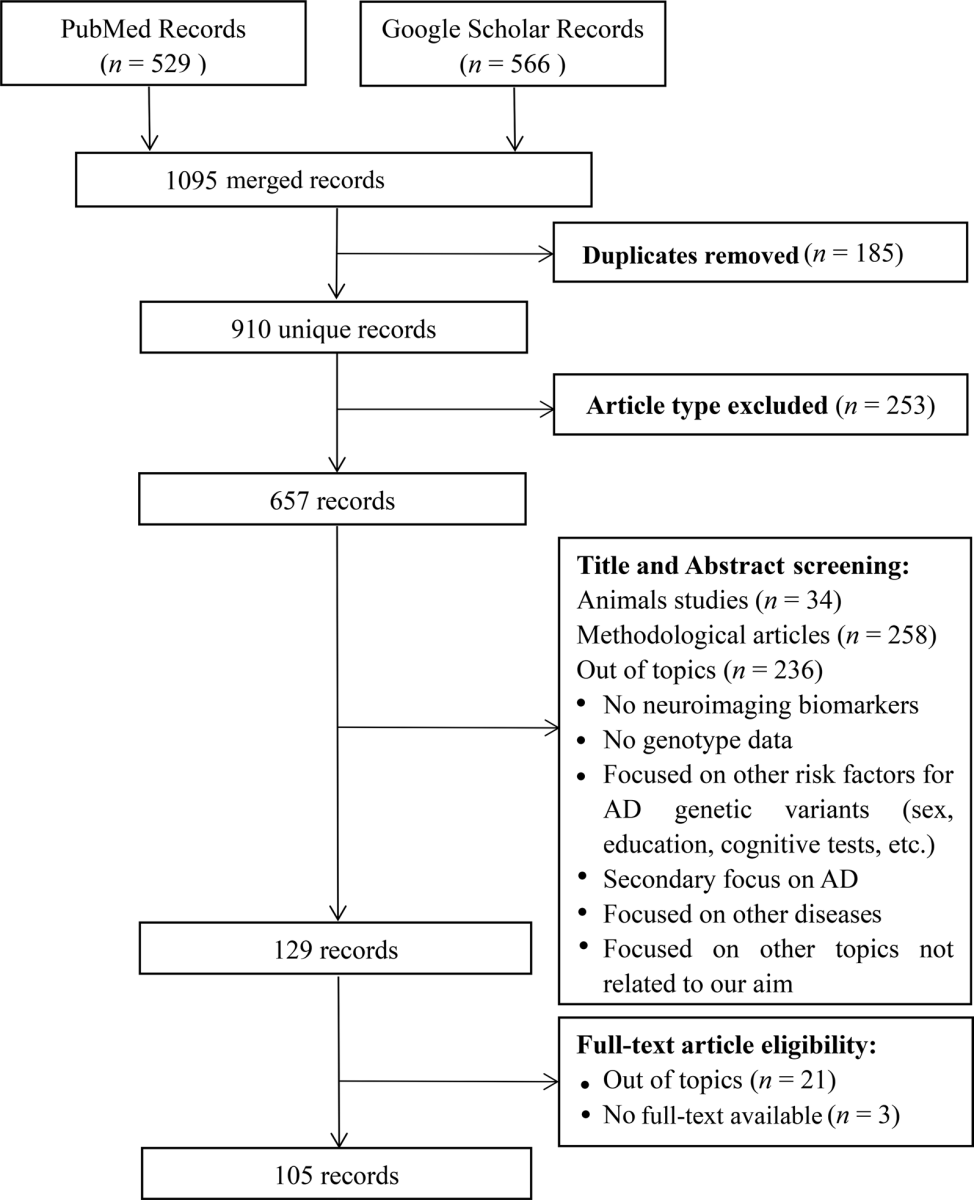
A flowchart of the search and screening process for articles included in this review

The inclusion criteria were as follows: (1) studies that identified AD candidate variants in large GWAS and meta-analyses, or described imaging biomarker genomics associations based on the ATN framework, such as genome-wide associations, polygenic scores analyses, AD classification diagnosis and prognosis, etc.; (2) studies focused on quantitative analysis of neuroimaging markers by using amyloid PET, tau PET, fluorodeoxyglucose (FDG) PET, anatomic MRI, or other MRI techniques including fMRI and DTI; (3) studies focused on single nucleotide polymorphism (SNP) genotype analysis. Articles were excluded if they were: (1) case reports, reviews, study-design protocols, books and documents, thesis, editorials, communications, opinion (methodological perspective) articles, and letters to the editors; (2) animal studies; (3) focused on methodological proposal and comparison, (4) not related to neuroimaging markers based on the ATN framework (e.g., various CSF biomarkers or EEG recording), or focused on other risk factors for AD (e.g., sex, education, cognitive tests). Finally, 105 records were included in this review. The detailed process of literature search and screening is presented in Fig. 2.

## Evolving technologies of brain imaging biomarker genomics

The research field of brain imaging biomarker genomics has been developing for two decades. Initially, twin-based and family-based genetic designs were used to calculate the heritability of measures derived from neuroimaging, such as brain volume [40–42], functional connectivity [43], and WM structure [44]. These studies have confirmed that the brain imaging measures have a moderate to strong genetic effect in AD [45], suggesting the potential value of brain imaging biomarker genomics studies in AD. In this section, we will introduce the evolving technologies in this field and describe the technical frameworks used in AD research from both genetic and imaging perspectives.

### Analytical procedures for AD imaging

**Fig. 3:**
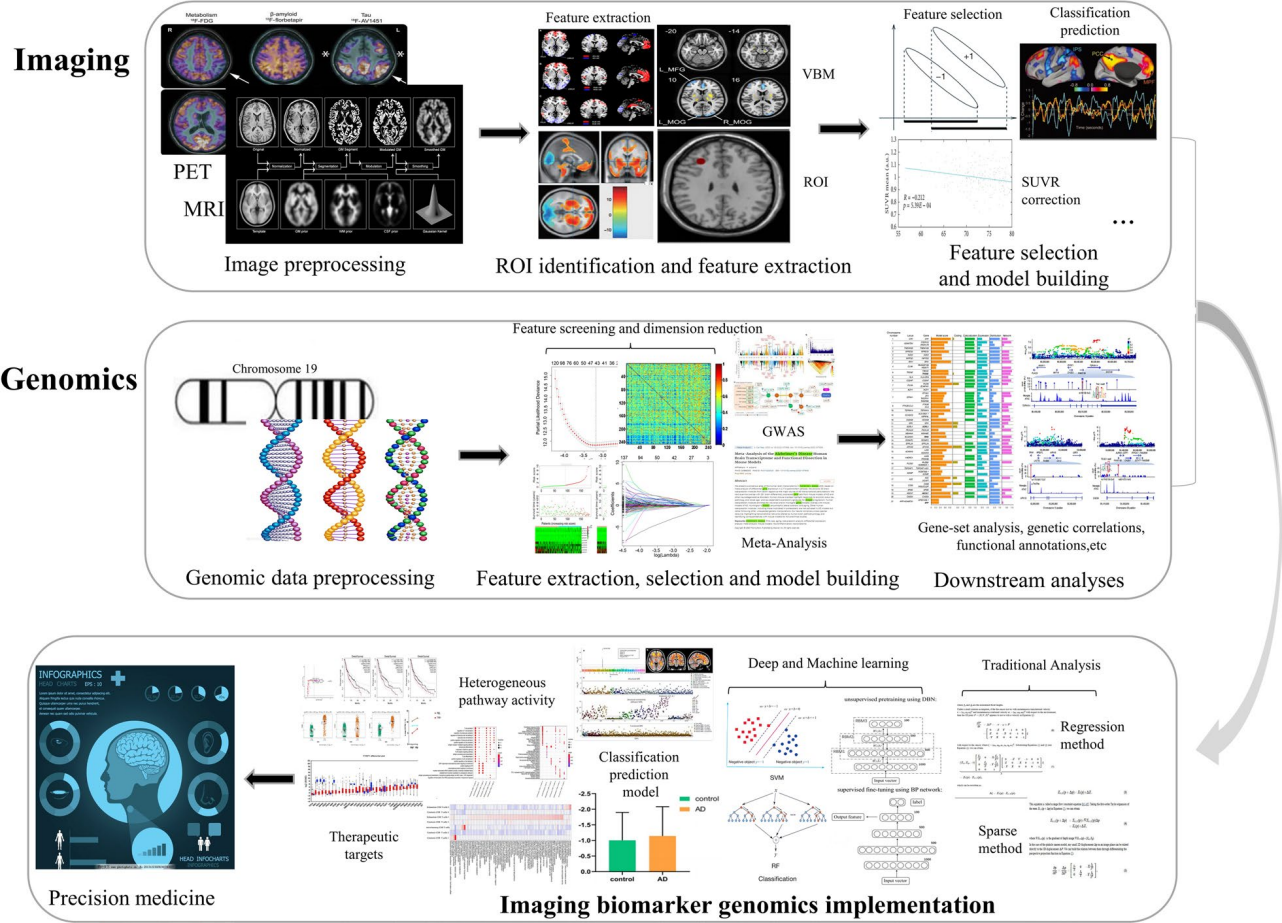
Systematic computational framework for studies in the field of AD brain imaging biomarker genomics. The top panel indicates the analytical steps involved in imaging: image preprocessing, identification of regions of interest, feature extraction, feature selection, and model building and evaluation. The middle panel represents genomics procedures: genetic preprocessing, feature extraction and dimension reduction, model building, and statistical analysis. The bottom panel indicates integrated analysis methods in studies of imaging biomarker genomics, including association analysis, classification and prediction

The systematic framework of brain imaging biomarker genomics for AD is composed of three panels: imaging, genomics and imaging biomarker genomics (Fig. 3).

Based on the ATN framework, the commonly used imaging techniques for AD are MRI and PET. MRI mainly includes sMRI, fMRI and DTI. PET imaging includes [^18^F] FDG PET, [^18^F] AV45 or [^11^C] Pittsburgh compound B ([^11^C] PiB) amyloid PET, and [^18^F] AV1451 tau-PET. Advances in imaging technologies have led to noninvasive or minimally invasive imaging of biomarkers, which may help capture all aspects of the disease process, including amyloid deposition [46], tau pathology [47], functional decline [48] and neuronal loss [49]. Below are the calculation frameworks for imaging analysis.

#### Step 1 Image preprocessing

High-resolution sMRI preprocessing includes realignment, segmentation, spatial normalization and smoothing. PET image processing includes realignment, coregistration, partial-volume correction, spatial normalization and smoothing. Resting-state fMRI preprocessing includes removal of unstable time points, slice timing corrections, head-motion corrections, baseline drift removal, spatial normalization and spatial smoothing. DTI data preprocessing includes skull stripping, background region filtering, and head-motion and eddy-current corrections. Several toolboxes can be used for this purpose, such as FSL (FMRIB’s Software Library) that processes MRI images (task or resting-state fMRI, sMRI, DTI, etc.) [50], Freesurfer that provides a series of algorithms to quantify brain functional and structural markers [51], and statistical parametric mapping (SPM) that is used for PET image preprocessing [52, 53]. More specifically, Data Processing & Analysis for Brain Imaging (DPABI) provides a complete resting-state fMRI analysis pipeline [54]. Other toolkits, such as DPARSF (Data Processing Assistant for Resting-State fMRI) and REST (Resting-State fMRI Data Analysis Toolkit) are also useful for fMRI analysis.

#### Step 2 Identification of regions of interest (ROIs) and feature extraction

This step includes precise identification of ROIs and extraction of imaging features [55, 56]. There are two common approaches to locating ROIs in brain imaging analyses: the voxel-based morphometry (VBM)-based method and the atlas-based method. VBM can achieve quantitative detection of differences in voxel-level imaging characteristics between groups. The atlas-based method projects the partitioning information from the standard brain atlas onto the images to identify specific brain regions. The identification of ROIs is followed by manual/automatic extraction of imaging features. The detailed characterization and calculation of imaging features are elaborated in Table 1. Feature extraction can usually be carried out by using FSL, Freesurfer, DPABI, SPM, the radiomics tool developed by Vallieres et al. (https://github.com/mvallieres/radiomics), and the Brain Connectivity Toolbox for graph theory-based brain network analyses [57].

**Table 1:** Summary of imaging radiomics features and calculation formulas

	Feature name	Calculation formula
First-order features	SUVR	$SUVR_{mean} = \frac{{I_{avg\_ROIC} }}{{I_{avg\_ref} }}$
	FA	$\sqrt {\frac{{(\lambda_{1} - \lambda_{2} )^{2} + (\lambda_{1} - \lambda_{3} )^{2} + (\lambda_{2} - \lambda_{3} )^{2} }}{{2(\lambda_{1} + \lambda_{2} + \lambda_{3} )^{2} }}}$
	Skewness	$\sigma^{ - 3} \mathop \sum \limits_{i = 1}^{{N_{g} }} \left( {i - \mu } \right)^{3} p\left( i \right)$
	Kurtosis	$\sigma^{ - 4} \mathop \sum \limits_{i = 1}^{{N_{g} }} [\left( {i - \mu } \right)^{4} p\left( i \right)] - 3$
	Variance	$\mathop \sum \limits_{i = 1}^{{N_{g} }} \left( {i - \mu } \right)^{2} p\left( i \right)$
	Other First-order features: cortical thickness; grey matter volume (sMRI features); ALFF, fALFF, ReHo, FC (fMRI signals); MD, radD, axD (DTI diffusion parameters); clustering coefficient, characteristic path length, small-worldness, global efficiency, transitivity, assortativity coefficient, modularity (various network parameters); and so on	
High-dimensional features	Energy	$\mathop \sum \limits_{i = 1}^{{N_{g} }} \mathop \sum \limits_{j = 1}^{{N_{g} }} \left[ {p\left( {i,j} \right)} \right]^{2}$
	Strength	$\frac{{\mathop \sum \nolimits_{i = 1}^{{N_{g} }} \mathop \sum \nolimits_{i = 1}^{{N_{g} }} \left( {n_{i} + n_{j} } \right)\left( {i - j} \right)^{2} }}{{\left[ {\varepsilon + \mathop \sum \nolimits_{i = 1}^{{N_{g} }} s\left( i \right)} \right]}},n_{i} \ne 0,n_{j} \ne 0$
	Entropy	$\mathop \sum \limits_{i = 1}^{{N_{g} }} \mathop \sum \limits_{j = 1}^{{N_{g} }} p\left( {i,j} \right)log\left( {p\left( {i,j} \right)} \right)$
	GLN	$\mathop \sum \limits_{i = 1}^{{N_{g} }} (\mathop \sum \limits_{j = 1}^{{N_{r} }} r\left( {i,j} \right))^{2}$
	LRHGE	$\mathop \sum \limits_{i = 1}^{{N_{g} }} \mathop \sum \limits_{j = 1}^{{N_{r} }} i^{2} j^{2} r\left( {i,j} \right)$
	GLV	$\frac{1}{{N_{g} \times N_{r} }}\mathop \sum \limits_{i = 1}^{{N_{g} }} \mathop \sum \limits_{j = 1}^{{N_{r} }} \left( {ir\left( {i,j} \right) - \mathop \sum \limits_{i = 1}^{{N_{g} }} i\mathop \sum \limits_{j = 1}^{{N_{r} }} r\left( {i,j} \right)} \right)^{2}$
	Other High-dimensional features are based on other analytical methods	

*ALFF* amplitude of low-frequency fluctuations, *axD* axial diffusivity, *FA* fractional anisotropy, *fALFF* fractional ALFF, *FC* functional connectivity, *GLN/GLV* grey-level non-uniformity/variance, *LRHGE* long run high grey-level emphasis, *MD* mean diffusivity, *radD* radial diffusivity, *ReHo* regional homogeneity, *SUVR* standard update value ratios. Where $I_{avg\_ROIC}$ is the average intensity of the brain regions, $I_{avg\_ref}$ is the average intensity of the reference region, $\lambda_{1} ,\lambda_{2} ,\lambda_{3}$ means the DTI eigenvalues, $N_{g}$ denotes the number of grey levels, $N_{r}$ is the maximum distance of run lengths, $p\left( i \right)$ denotes the number of pixels with grey level $i$ in the normalized grey histogram, and $\mu$ denotes the mean value

#### Step 3 Feature selection and model building

The aims of feature selection are to reduce feature redundancy and remove irrelevant features. Common feature selection methods include consistent stability analysis, statistical tests (two-sample *t*-test and rank-sum test), correlation analysis, sparse-group lasso, etc. There are two types of model construction: classification/prediction models and other statistical analysis models, such as regression analysis, correlation analysis, and survival analysis. Finally, model generalization capabilities are evaluated in terms of accuracy, sensitivity, specificity, correlation coefficient, and regression coefficient.

The above processes could also be carried out using deep learning (DL) algorithms, which can automatically extract quantitative and high-throughput features from medical images by end-to-end deep neural networks, which avoids complex hand-coding and does not need prior knowledge [58–61].

### Analytical procedures for AD genomics

Early studies of brain genomics mainly focused on linkage and association analyses [62], in which candidate genetic markers were selected typically based on a hypothesis that implicates certain genes in AD pathogenesis. Advances in large-scale genotyping technologies enable comprehensive, unbiased GWAS, which can simultaneously test thousands of genetic markers. Nevertheless, GWAS might not avoid statistical artefacts that arise from the large number of tests. Systematic meta-analysis can alleviate this situation because this approach can quantitatively synthesize published genotype data for each polymorphism and produce a summary risk estimate (called the odds ratio) that contributes to the overall interpretation of association studies independent of positive or negative outcomes. Moreover, with the increase of sample sizes in GWAS analyses, ploygenic scores (PGS) are emerging as a novel statistical index that associates the collective individual SNP genotypes with specific diseases [63, 64]. In summary, AD genomics studies are mainly concentrated on traditional linkage and association analyses, large-scale case–control GWAS, systematic GWAS meta-analyses and recent PGS analyses, which facilitate identification of novel AD susceptibility genes as well as early diagnosis and prevention. The calculation frameworks for genomic analysis are mainly as follows.

#### Step 1 Genomic data preprocessing

As the first step, genomic data preprocessing includes quality control and imputation of genotyping data. Standard genotyping data quality control at the sample and variant level can be performed following a previously published pipeline [65, 66]. Genotyping data imputation is performed based on the Haplotype Reference Consortium (full panel) and the 1000 Genomes reference panel (for indels only).

#### Step 2 Feature extraction, selection and model building

This step aims at data mining and statistical analysis. Data mining focuses on feature extraction and dimensionality reduction, and constructs classification/prediction and statistical models with consideration of the complex nature of large genomics data. Statistical analysis mainly refers to construction of threshold-based association analysis models, including GWAS and meta-analysis. Subsequently, replication studies are always conducted to validate the results.

#### Step 3 Downstream analyses

Downstream analyses include conditional analysis, statistical fine-mapping analysis, colocalization with expression quantitative trait loci and metabolism quantitative trait loci, functional annotation, network analysis, gene-based analysis, gene set or tissue enrichment analysis, linkage disequilibrium analysis, PGS analysis, gene pleiotropy, heritability, genetic correlation calculation, etc.

### Analytical procedures for AD imaging biomarker genomics

In general, the research field of AD imaging biomarker genomics is mainly focused on univariate or multivariate association analyses using imaging phenotypes as an intermediate. For example, Kim et al. [67] investigated genetic variants that influence cortical atrophy in 919 participants from the Alzheimer’s Disease Neuroimaging Initiative (ADNI) database. They analyzed correlations between 3,041,429 SNPs selected based on GWAS and cortical thickness in the whole brain. This study included three steps: (1) imaging/genomic data preprocessing; (2) calculation of cortical thickness as an imaging feature; and (3) statistical analysis. The results of the study identified that rs10109716 in *ST18* and rs661526 in *NFIA* are significantly associated with the mean cortical thicknesses of the left inferior frontal gyrus and left parahippocampal gyrus, respectively. In addition, Ning et al. [68] employed a neural network (NN) framework that combined both brain atrophic measurements and SNP genotype data to distinguish AD patients from healthy controls (HC). In this study, volumes of 16 ROIs selected based on prior knowledge on brain regions affected by AD were used as the imaging feature, and genotypes of *APOE* ɛ4 risk allele and 19 SNPs were used as the genetic features. The results showed that the NN model with both imaging and genetic features had an area under the receiver operating characteristic curve (AUC) of 0.99 in classifying AD and HC subjects.

## Implementation of AD imaging biomarker genomics studies

### Findings from studies on candidate genetic variants for AD

**Fig. 4:**
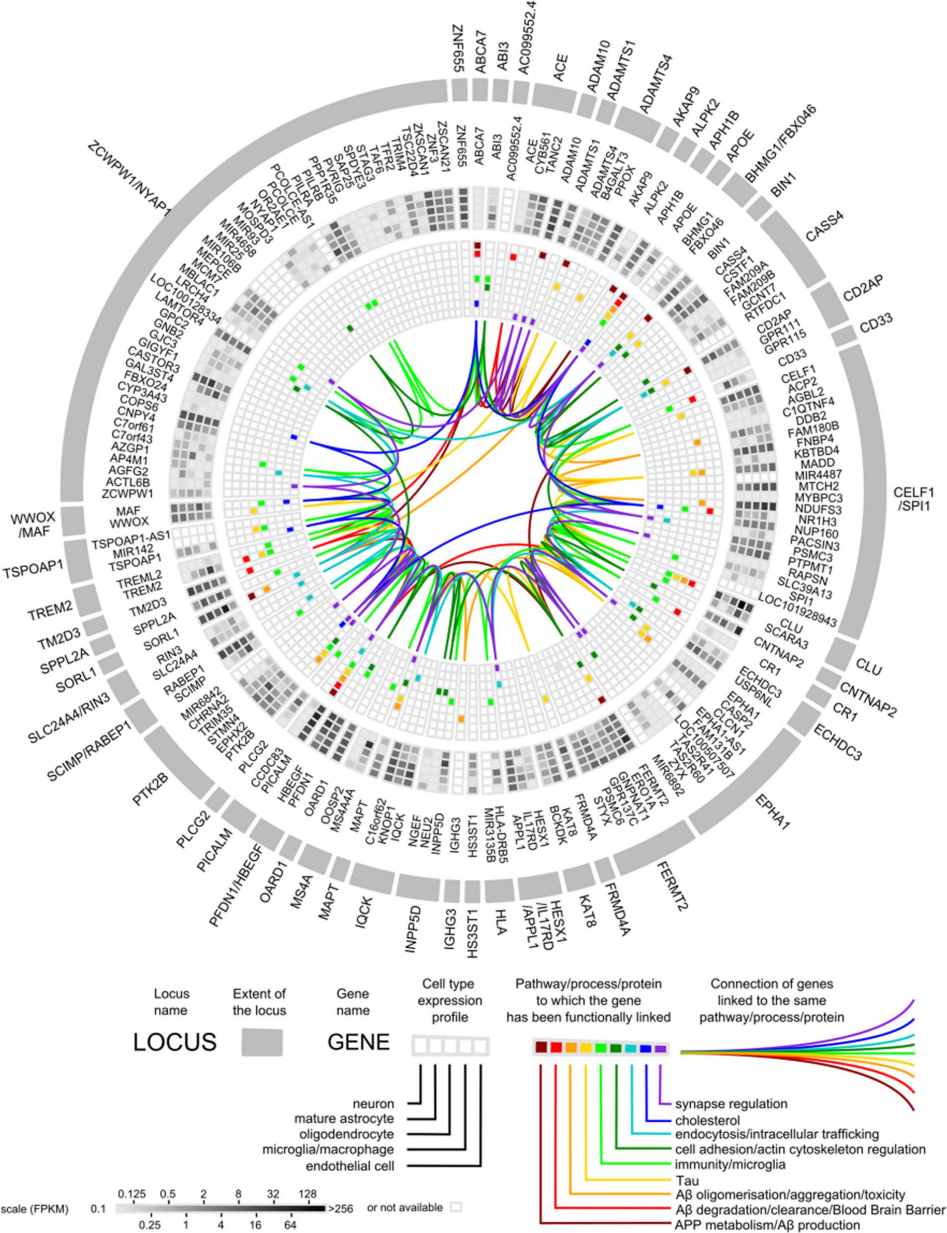
Circular diagram of AD genetic risk factors. From outside to inside: (1) genomic loci in alphabetical order; (2) genes therein; (3) expression profiles of these genes in different cell types of the brain (greyscale); and (4) pathways/processes/proteins to which these genes have been functionally linked (colour lines). Adapted from Dourlen P et al. Acta Neuropathologica. 2019 Aug; 138 (2):221–236. Reprinted with permission from Springer Nature

Since imaging biomarker genomics studies rely in part on  prior knowledge of candidate genetic variants, we summarize the candidate variants in accordance with the timeline of identification in large GWAS and meta-analyses. Initially, mutations of *APP*, *PSEN1* and *PSEN2* genes were found in molecular studies in 1993 and in 1995, which caused rare, Mendelian forms of the disease, usually resulting in early-onset AD. *APOE* was recognized as the strongest susceptibility gene for late-onset AD (LOAD) in 1995. In studies to confirm new risk loci related to LOAD, GWAS and meta-analyses further identified a series of loci relevant to LOAD. The first GWAS study was conducted in 2007. Later, GWAS studies were separately performed in four LOAD genetic consortia (Genetic and Environmental Risk in Alzheimer’s Disease, European Alzheimer’s Disease Initiative, Cohorts for Heart and Aging Research in Genomic Epidemiology, and Alzheimer’s Disease Genetic Consortium), which identified a total of 11 loci, namely, *CLU*, *PICALM*, *CR1*, *BIN1*, *CD2AP*, *CD33*, *EPHA1*, *MS4A4A*, *ABCA7*, *MS4A6A*, and *MS4A4E* [16, 27–30]. Under the support from the International Genomics of Alzheimer’s Project (IGAP), a meta-analysis including 74,046 individuals of European ancestry further identified 11 new susceptibility loci for AD, which were *HLA-DRB5*, *SORL1*, *PTK2B*, *SLC24A4-RIN3*, *ZCWPW1*, *NME8*, *FERMT2*, *CELF1*, *INPP5D*, *MEF2C* and *CASS4* [31]. A case–control study of 85,133 subjects from the IGAP identified 3 rare coding variants in *PLCG2*, *ABI3*, and *TREM2*, which are highly expressed in microglia, highlighting the contribution of microglial-mediated innate immunity to the development of AD [32]. Given the difficulty of AD case confirmation, a case–control genome-wide association study by proxy (GWAX) was conducted with the UK Biobank dataset using family history of disease (14,482 proxy cases, i.e., relatives of affected individuals and 10,0082 proxy controls, i.e., relatives of unaffected individuals). Meta-analysis of the previously published IGAP GWAS results combining with the above-highlighted GWAX summary statistics identified 4 new risk loci associated with AD (*HBEGF*, *ECHDC3*, *SPPL2A*, and *SCIMP*) [33]. In the following year, a second meta-analysis of IGAP data and parental history of AD in an expanded UK Biobank dataset (*n* = 314,278) based on the previous proxy-phenotype AD study by Liu et al*.* identified 3 new loci (*ADAM10*, *KAT8*, and *ACE*) [34]. A larger meta-analysis with clinically diagnosed AD and AD-by-proxy (71,888 cases, 383,378 controls), using cohorts collected by the Psychiatric Genomics Consortium Alzheimer, the IGAP, the Alzheimer’s Disease Sequencing Project and AD-by-proxy from UK Biobank, yielded 8 loci (*ADAMTS4*, *HESX1*, *CLNK*, *CNTAP2*, *APH1B*, *ABI3*, *ALPK2*, and *ACO74212.3*) [21]. An expanded IGAP analysis (*n* = 94,437) confirmed 20 previous LOAD risk loci and identified 5 new loci (*IQCK*, *ACE*, *ADAM10*, *ADAMTS1* and *WWOX*) [20], two of which (*ACE* and *ADAM10*) had been recently identified in the study of Marioni et al*.* [34]. Following the meta-analysis of Lambert et al*.* and Marioni et al*.*, an updated meta-analysis of GWAX in the UK Biobank with the latest GWAS for AD diagnosis was performed and identified 37 risk loci and 4 new associations (*CCDC6*, *TSPAN14*, *NCK2* and *SPRED2*) [24]. Finally, the most recent GWAS with 1,126,563 individuals, which expanded on the basis of Jansen’s work and contained the largest sample size thus far, identified 38 loci, including 7 loci (*AGRN*, *TNIP1*, *TMEM106B*, *GRN*, *HAVCR2*, *NTN5* and *LILRB2*) that had not been reported previously [25]. A detailed summary of the representative AD candidate genes is shown in Table 2. Figure 4 depicts a circular diagram of AD genetic risk factors according to several postgenomics analyses based on animal and cellular models, although the AD genetic background remains largely unidentified.

**Table 2:** Summary of candidate genes used in AD pathology

Year	Author	Dataset	Methods	Novel genes
1991 [7]	Goate et al.	Gene Cloning	Molecular studies	*APP* gene
1993 [10]	Corder et al.	Gene Cloning	Molecular studies	*APOE* gene
1995 [8, 9]	Sherrington et al.	Gene Cloning	Molecular studies	2 genes (*PSEN1* and *PSEN2*)
2009–2011 [16, 27– 30]	Lambert et al.	GERAD EADI CHARGE ADGC	Meta-analysis	11 genes (*CLU*, *PICALM*, *CR1*, *BIN1*, *CD2AP*, *CD33*, *EPHA1*, *MS4A4A*, *ABCA7*, *MS4A6A*, and *MS4A4E*)
2013 [31]	Lambert et al.	IGAP (*n* = 74,046)	Meta-analysis	11 genes (*HLA-DRB5*, *SORL1*, *PTK2B*, *SLC24A4-RIN3*, *ZCWPW1*, *NME8*, *FERMT2*, *CELF1*, *INPP5D*, *MEF2C, *and *CASS4*)
2017 [32]	Sims et al.	IGAP (*n* = 85,133)	Meta-analysis	3 genes (*PLCG2*, *ABI3*, and *TREM2*)
2017 [33]	Liu et al.	UK Biobank (*n* = 116,196)	Meta-analysis	4 genes (*HBEGF*, *ECHDC3*, *SPPL2A*, and *SCIMP*)
2018 [34]	Marioni et al.	UK Biobank (*n* = 314,278)	Meta-analysis	3 genes (*ADAM10*, *KAT8*, and *ACE*)
2019 [21]	Jansen et al.	PGC-ALZ IGAP ADSP (*n* = 455,266)	Meta-analysis	8 genes (*ADAMTS4*, *HESX1*, *CLNK*, *CNTAP2*, *APH1B*, *ABI3*, *ALPK2*, and *ACO74212.3*)
2019 [20]	Kunkle et al.	IGAP (*n* = 94,437)	Meta-analysis	5 genes (*IQCK*, *ACE*, *ADAM10*, *ADAMTS1*, and *WWOX*)
2020 [24]	Schwartzentruber et al.	UK Biobank (*n* = 408,942)	Meta-analysis	4 genes (*CCDC6*, *TSPAN14*, *NCK2*, and *SPRED2*)
2021 [25]	Wightman et al.	1,126,563 individuals	Meta-analysis	7 genes (*AGRN*, *TNIP1*, *TMEM106B*, *GRN*, *HAVCR2*, *NTN5*, and *LILRB2*)

### Findings from studies on AD candidate imaging biomarkers

In earlier studies, pairwise univariate analysis was performed to identify associations between genetic markers and imaging phenotypes. To accommodate more flexible associations involving multiple genetic markers and multiple imaging phenotypes, multiple regression and multivariate models have been used in recent studies in combination with machine learning (ML) methods [69]. In the following, we will review candidate-gene, genome-wide and polygenic associations with imaging-derived traits, according to the ATN framework for AD biomarkers proposed by NIA-AA in 2018 (Table 3) [39].

**Table 3:** Summary of AD-relevant effects based on candidate imaging biomarkers and association studies

Author	Dataset	Genes included	Model	Methods	Imaging phenotypes	Neural location	Results
**Pathophysiological pathway: Brain Aβ accumulation (Aβ PET)**							
2009 Drzezga et al. [70]	32 AD	*APOE*	Univariate imaging—Univariate genetic	Candidate-based association	Aβ plaque deposition	Bilateral temporoparietal, frontal cortex	The ɛ4-positive patients with AD had higher levels of Aβ plaque deposition compared to age-matched ɛ4-negative patients with similar levels of cognitive impairment and brain atrophy
2009 Reiman et al. [71]	28 AD	*APOE*	Univariate imaging—Univariate genetic	Candidate-based association	PiB DVR fibrillar Aβ burden	Frontal, temporal, parietal, posterior cingulate-precuneus,basal ganglia ROIs	Fibrillar Aβ burden in cognitively normal older people was associated with APOE ɛ4 gene dose
2011 Chibnik et al. [72]	*n* = 1666	*CR1*, *CLU*, *PICALM*	Univariate imaging—Multivariate genetic	Candidate-based association	Pathology score of neuritic plaques	Whole brain cortex	Common variation at the *CR1* locus had a broad impact on cognition and this effect was mediated by an individual’s amyloid plaque burden
2012 Thambisetty et al*.* [73]	57 HC	*CR1*, *APOE*	Univariate imaging—Multivariate genetic	Candidate-based association	PIB DVR	Orbitofrontal, prefrontal, superior frontal, posterior cingulate, lateral temporal, occipital cortices	There was a greater variability in brain amyloid deposition in the *CLU* rs3818361 noncarrier group relative to risk carriers, an effect explained partly by *APOE* genotype
2012 Swaminathan et al. [74]	ADNI (22 HC, 25 AD, 56 MCI)	15 amyloid candidate genes (*DNCR24*, *NCSTN*, *SOAT1*, *BCHE*, etc*.*)	Multivariate imaging—Multivariate genetic	Candidate-based association	Normalized PiB uptake value	Anterior cingulate, frontal cortex, parietal cortex, precuneus	The minor allele of an intronic SNP within *DHCR24* was identified and associated with a lower average PiB uptake, and non-carriers of the minor allele had higher PiB uptake in frontal regions compared to carriers
2013 Shulman et al. [75]	Multiple cohorts (*n* = 725/ 56/58)	*ABCA7*, *MS4A6A/MS4A4E*, *EPHA1*, *CD3*, *CR1*, *CD2AP*, *CLU*, *BIN1*, *PICALM*	Univariate imaging—Multivariate genetic	Candidate-based association	Pathology score of neuritic plaques	Midfrontal, middle temporal, inferior parietal, entorhinal, hippocampal cortex	Besides the previously reported *APOE* and *CR1* loci, *ABCA7* (rs3764650) and *CD2AP* (rs9349407) were associated with neuritic plaque burden
2013 Shulman et al. [75]	Multiple cohorts (*n* = 725/ 56/58)	Genome-wide genotyping	Univariate imaging—Multivariate genetic	GWAS	Pathology score of neuritic plaques	Midfrontal, middle temporal, inferior parietal, entorhinal, hippocampal cortex	The finding discovered a novel variant near the amyloid precursor protein gene (*APP*, rs2829887) that is associated with neuritic plaques
2013 Hohman et al. [76]	ADNI (174 HC, 64 AD, 292 MCI)	*PICALM*, *BIN1*, *CR1*, *CLU*, *MS4A6A*, *EPHA1*, *CD33*, *ABCA7*, *CD2AP*	Multivariate imaging—Univariate genetic	Candidate-based association	Aβ PET SUVR	Cingulate, frontal, temporal, lateral parietal cortices	Two SNP-SNP interactions (*BIN1* (rs7561528, rs744373) × *PICALM* (rs7851179)) reached significance when correcting for multiple comparisons
2014 Lehmann et al. [77]	52 AD	*APOE*	Multivariate imaging—Univariate genetic	Candidate-based association	PIB DVR, FDG SUVR	Frontal, lateral parietal/temporal, occipital cortices, precuneus, posterior cingulate gyrus, hippocampus	*APOE* ε4+ AD patients showed lower global amyloid burden and greater medial temporal hypometabolism compared with matched *APOE * ε4- patients
2014 Ramanan et al. [78]	ADNI (*n* = 555)	Genome-wide genotyping	Univariate imaging—Multivariate genetic	GWAS	Aβ PET brain amyloid burden	Frontal, parietal, temporal, limbic, occipital lobes	A novel association with higher rates of amyloid load independent from *APOE * ε4 status was identified in *IL1RAP* (rs12053868-G)
2018 Apostolova et al. [17]	ADNI (322 HC, 159 AD, 496 MCI)	The top 20 AD risk variants (*ABCA7*,*CLU*, *SORL1*, *DSG2*, etc.)	Univariate imaging—Multivariate genetic	Candidate-based association	Florbetapir mean SUVR	Frontal, anterior–posterior cingulate, lateral-parietal, lateral-temporal cortices	*ABCA7* gene had the strongest association with amyloid deposition, after *APOE* ε4. *FERMT2* gene had a stage-dependent association with brain amyloidosis
2018 Scelsi et al. [79]	ADNI (226 HC, 125 AD, 92 SMC, 501 MCI)	Genome-wide genotyping	Multivariate imaging—Multivariate genetic	PGS-based association	Aβ PET SUVR, HV	Hippocampus	The finding identified a genome-wide significant locus implicating *LCORL* rs6850306. The possession of a minor allele at rs6850306 was protective against conversion from MCI to AD
2019 Li et al. [80]	ADNI (155 HC, 125 AD, 72 SMC, 422 MCI)	Genome-wide genotyping	Univariate imaging—Multivariate genetic	GWAS	Florbetapir composite SUVR	Frontal, anterior/ posterior cingulate, lateral parietal/ temporal regions	The study identified 24 consensus modules enriched by robust genetic signals in a genome wide association analysis, including a few novel genes (*ABL1*, *ABLIM2*)
2021 Kim et al [81]	Korean cohort (*n* = 1474)	Genome- wide genotyping	Univariate imaging—multivariate genetic	GWAS	Aβ PET SUVR	Whole brain	In addition to *APOE*, nine SNPs of *FGL2* gene on chromosome 7 were identified, which were associated with a decreased risk of Aβ positivity at a genome-wide suggestive level
2021 Liu et al. [82]	Multiple cohorts (*n* = 767/ 1373)	Summary statistics	Multivariate imaging—Multivariate genetic	PGS-based association	Aβ PET SUVR, HV, entorhinal, middle temporal gyrus volumes	Whole brain cortex, Hippocampus, entorhinal cortex	PGS was associated with the increased cortical amyloid burdens (PiB-PET and AV45-PET), but decreased hippocampus and entorhinal cortex volumes
**Pathophysiological pathway: Tau hyperphosphorylation (Tau PET)**							
2016 Smith et al. [83]	4 HC, 3 AD	*MAPT*	Univariate imaging—Univariate genetic	Candidate-based association	Tau PET SUVR, GM volume	Global AD pathology	^18^F-AV1451 tau PET imaging correlated with tau pathology in *MAPT* mutation carriers
2018 Mattsson et al. [84]	65 Aβ + patients	*APOE*	Univariate imaging—Univariate genetic	Candidate-based association	Tau PET SUVR, GM volume	Parietal, entorhinal cortex	*APOE* ε4-negative patients had greater tau load and reduced cortical thickness, with the most pronounced effects for both in the parietal cortex
2019 Shen et al. [85]	ADNI (90 HC)	*MAPT* rs242557	Univariate imaging—Univariate genetic	Candidate-based association	Tau PET SUVR	Hippocampus	The finding confirmed the significant correlation of *MAPT* rs242557 risk variant with increased hippocampus tau burden in non-demented elders
2019 Therriaultet al. [86]	Multiple cohorts (281 HC, 75 AD, 133 MCI)	*APOE*	Univariate imaging—Univariate genetic	Candidate-based association	Tau PET SUVR	Entorhinal cortex, hippocampus	The elevated risk of developing dementia conferred by *APOE* ε4 genotype involved mechanisms associated with both Aβ and tau aggregation
2019 Franzmeier et al. [87]	ADNI (49 HC, 40 MCI)	*BIN1* rs744373	Univariate imaging—Univariate genetic	Candidate-based association	Global/stage- specific Tau PET SUVR	Brain Braak stage II–VI	*BIN1* rs744373 SNP was associated with increased tau but not Aβ pathology, that is alterations in *BIN1* may contribute to memory deficits via increased tau pathology
2020 Yan et al. [88]	ADNI (57 AD)	*APOE*	Multivariate imaging—Univariate genetic	Candidate-based association	Tau PET SUVR, GM volume	Temporal, parietal, posterior cingulate, entorhinal cortex, amygdala, parahippocampal gyrus, etc	Among elderly individuals with AD, sex modified the effects of the *APOE* ε4 allele on region-specific tau deposition and GM volume
2020 Neitzel et al. [89]	Multiple cohorts (*n* = 493)	*APOE*	Univariate imaging—Univariate genetic	Candidate-based association	Baseline Tau PET SUVR, annual change rates	MTL (entorhinal cortex, parahippocampus)	There was an amyloid-independent association between *APOE* ε4 and elevated tau PET specifically in medial temporal regions
2021 Franzmeier et al. [90]	Multiple cohorts (*n* = 216)	*BIN1* rs744373	Univariate imaging—Univariate genetic	Candidate-based association	ROI Tau PET SUVR, annual change rates	Whole brain	*BIN1*-associated AD risk was potentially driven by accelerated tau accumulation in the face of Aβ
2021 Neitzel et al. [91]	ADNI (347 HC, 48 AD, 156 MCI)	Klotho-VS^het^	Multivariate imaging—Univariate genetic	Candidate-based association	Global/ROI tau/Aβ PET SUVR	Whole brain; bilateral inferior temporal gyri	Findings proved a protective role of KL-VS^het^ against amyloid-related tau pathology and tau-related memory impairments in elderly humans at risk of AD dementia
2021 Sun et al. [92]	ADNI (*n* = 158)	Summary statistics	Multivariate imaging—Multivariate genetic	PGS-based association	Global tau SUVR for Braak stage ROIs	Whole brain	The association between PGS and tau pathology was significant when *APOE* was excluded, even among females
**Pathophysiological pathway: Neurodegeneration (sMRI)**							
2007 Lunetta et al. [93]	449 HC, 366 AD	*APOE*	Univariate imaging—Univariate genetic	Candidate-based association	Cerebral atrophy, MTA, WMH, CVR	Cerebral atrophy, MTA, WMH	A substantial proportion of the additive genetic variation in MRI traits was explained by other genes, and MRI traits were heritable
2009 Potkin et al. [94]	ADNI (*n* = 381)	Genome-wide genotyping	Univariate imaging—Multivariate genetic	GWAS	GM voxels of hippocampal regions	The right and left hippocampal regions	The study identified candidate risk genes (*EFNA5*, *CAND1*, *MAGI2*, *ARSB*, and *PRUNE2*) for sporadic AD, involved in the regulation of protein degradation, apoptosis, neuronal loss and neurodevelopment
2010 Wolk et al. [95]	ADNI (91 AD)	*APOE*	Univariate imaging—Univariate genetic	Candidate-based association	Cortical thickness, HV	Hippocampus, superior frontal gyrus,angular gyrus, MTL, precentral gyrus	The presence or absence of the* APOE* ε4 allele influenced the cognitive and anatomic phenotypic expression of AD in a dissociable manner
2010 Biffi et al. [96]	Multiple cohorts (215 HC, 168 AD, 357 MCI)	GWAS-validated and GWAS-promising novel AD loci	Univariate imaging—Multivariate genetic	Candidate-based association	HV, amygdala volume, WM lesion volume, parahippocampal, entorhinal, temporal pole cortex thickness	Hippocampal, parahippocampal gyrus, amygdala, entorhinal, temporal pole cortex	Loci associated with AD influenced neuroimaging correlates of this disease. And neuroimaging analysis identified 2 additional loci (*BIN1* and *CNTN5*) of high interest for further study
2013 Meda et al. [97]	ADNI (156 HC, 140 AD, 281 MCI)	151 million SNPs within 212 KEGG pathways	Univariate imaging—Multivariate genetic	Candidate-based association	12-month regional structural atrophy rates	Hippocampus, entorhinal cortex	A total of 109 SNP-SNP interactions were associated with right hippocampus atrophy, and 125 were associated with right entorhinal cortex atrophy
2013 Jahanshad et al. [98]	366 HC	*SPON1* gene	multivariate imaging—multivariate genetic	Candidate-based association	Heritable brain connections	Maps of the brain’s structural connectome	Rs2618516 was shown to affect brain structure in an elderly population with varying degrees of dementia
2014 Morgen et al. [99]	165 AD	*PICALM*, *APOE*	Univariate imaging—Multivariate genetic	Candidate-based association	GM volume	Prefrontal cortex	There was a synergistic adverse effect of homozygosity for the *PICALM* risk allele G in rs3851179 and *APOE * ε4 on prefrontal volume and performance on the Trail Making Test A, which is sensitive to processing speed and working memory function
2014 Hohman et al. [100]	ADNI (388 HC, 228 AD, 764 MCI)	Genome-wide genotyping	Univariate imaging—multivariate genetic	GWAS	Baseline ICV	Whole brain	One intergenic SNP rs4866650 and one SNP rs7849530 within the *SPTLC1* gene modified the association between amyloid positivity and neurodegeneration
2015 Chauhan et al. [101]	8175– 11,550 HC	24 AD candidate loci (*APOE*, *BIN1*,*HLA-DRB1*,*CR33*,*CR1*,*CLU*, *ABCA7*, *SORL1*, etc*.*)	Multivariate imaging—Multivariate genetic	Meta- analysis	ICV, TBV, HV, WMH	Hippocampus	*APOE* rs2075650 was associated with smaller HV and *CD33* rs3865444 with smaller ICV. There was associations of *HLA-DRB1* with TBV and *BIN1* with HV. A weighted AD genetic risk score was associated with smaller HV, even after excluding *APOE* locus
2015 Desikan et al. [102]	9386 HC, 6409 AD	Summary statistics	Univariate imaging—Multivariate genetic	PGS-based association	Longitudinal volume loss in MTL, entorhinal cortex, hippocampus	MTL, hippocampus, entorhinal cortex	Polygenic hazard scores predicted in vivo markers (volume loss in MTL, hippocampus, entorhinal cortex)
2016 Yang et al. [103]	ADNI (194 HC, 168 AD, 337 MCI)	*PICALM*, *CLU*	Univariate imaging—Multivariate genetic	Candidate-based association	HV, hippocampal shape	Hippocampus	Common LOAD risk loci in *CLU* and *PICALM* exhibited significant interaction effects on hippocampal morphology in both young healthy adults and elderly individuals
2016 Ramirez et al. [104]	50 HC, 98 MCI	the top 10 AD non-*APOE* genes	Univariate imaging—Multivariate genetic	Candidate-based association	Cortical thickness, hippocampal radial distance	Hippocampus	*MS4A6A* rs610932 and *ABCA7* rs3764650 demonstrated significant associations with cortical and hippocampal atrophy
2016 Habes et al. [105]	*n* = 1472	*APOE*	Univariate imaging—Univariate genetic	Candidate-based association	AD-related GM volume	Lateral frontal, lateral temporal, medial frontal cortex, hippocampus	Measurable *APOE*-related brain atrophy did not occur in early adulthood and midlife and such atrophy may only occur more proximal to the onset of clinical symptoms of dementia
2016 Foley et al. [106]	*n* = 272	*APOE*, summary statistics	Multivariate imaging—Multivariat genetic	PGS-based association	HV	Hippocampus	A significant association was found between AD PGS and left HV, with higher risk associated with lower left HV, although excluding the *APOE* gene
2016 Harrison et al. [107]	*n* = 66	Summary statistics	Univariate imaging—Multivariate genetic	PGS-based association	Thickness in hippocampal subregions	Hippocampus, entorhinal cortex	Polygenic AD risk scores may be especially sensitive to structural change over time in regions affected early in AD, like the hippocampus and adjacent entorhinal cortex
2017 Wang et al. [108]	ADNI (281 HC, 48 AD, 483 MCI)	12 SNPs in *HLA*	Univariate imaging—Multivariate genetic	Candidate-based association	Structural volumes	Hippocampus, parahippocampus, posterior cingulate, middle temporal, etc	*TNF-α* SNPs at rs2534672, rs2395488, *HFE* rs1800562 and *RAGE* rs2070600 were correlated with various structures on MRI
2017 Wang et al. [109]	ADNI (281 HC, 48 AD, 483 MCI)	*HLA-A2*	Univariate imaging—Univariate genetic	Candidate-based association	Hippocampal/ parahippocampal/ amygdala/ middle temporal/ posterior cingulate volume, entorhinal cortex thickness	Hippocampus, parahippocampus, posterior cingulate, precuneus, middle temporal, entorhinal cortex, amygdala	*HLA-A2* in Caucasians contributed to the risk of AD by modulating the alteration of HV and *HLA-A* gene variants appeared to play a role in altering AD-related brain structures on MRI
2017 Xiao et al. [110]	*n* = 231	*APOE*, summary statistics	Univariate imaging—Multivariate genetic	PGS-based association	Activation in hippocampus ROI	Hippocampus	There was a cumulative deleterious effect of LOAD risk genes on hippocampal function even in healthy volunteers
2018 Axelrud et al. [111]	Multiple cohorts	Summary statistics	Univariate imaging—Multivariate genetic	PGS-based association	HV	Left and right hippocampus	Genetic risk for AD may affect early-life cognition and HV
2018 Li et al. [112]	Multiple cohorts (*n* = 683)	Summary statistics	Univariate imaging—Multivariate genetic	PGS-based association	GM volume	Precuneal cortex	An elevated AD PGR was associated with a smaller precuneal volume, and the effect remained after excluding the *APOE* genotype
2019 Lancaster et al. [113]	Multiple cohorts	AD SNPs within a microglia-mediated immunity network	Univariate imaging—Multivariate genetic	PGS-based association	HV	Hippocampus	The observations suggested that the relationship between AD and HV was partially explained by genes within an AD-linked microglia-mediated immunity network
2020 Lyall et al. [114]	UK Biobank (*n* = 8539)	*APOE*	Multivariate imaging—Univariate genetic	Candidate-based association	FA, MD, left/right HV, total GM, total WM and log WMHV	Left or right Hippocampus, total GM and WM	There was association between *APOE * ε4 and WMHV, but not TBV or WM integrity
2020 Cong et al. [115]	ADNI (41 HC, 26 AD, 67 MCI)	Genome- wide genotyping	Univariate imaging—Multivariate genetic	GWAS	14 MTL substructures	MTL	A novel association with right Brodmann area 36 volume was discovered in an ERC1 SNP rs2968869. And rs2968869 was associated with GM density and glucose metabolism in the right hippocampus and disease status
2020 De Marco et al. [116]	ADNI (317 HC, 562 MCI)	Summary statistics	Univariate imaging—Multivariate genetic	PGS-based association	GM and WM volumes	Whole brain	PGS predicted volume in sensorimotor regions in ε3ε3 Aβ + participants. The link between polygenic hazard and neurocognitive variables varies depending on *APOE* ε4 allele status
2020 van der Meer et al. [117]	Multiple cohorts (*n* = 21,297)	Genome-wide genotyping	Univariate imaging—multivariate genetic	GWAS	Hippocampal and subfield volumes	Hippocampus	GWAS of whole HV identified eight whole-genome significant loci, including three novel loci, namely, *TFDP2* SNP rs7630893, *FAM175B* rs2303611, and *PARP11* rs1419859
2021 Foo et al. [118]	UK Biobank (*n* = 17,161)	Summary statistics	Univariate imaging—Multivariate genetic	PGS-based association	Volumes in hippocampal subregions	Multiple hippocampal regions	PGS_AD_ had differential effects on the hippocampal subfield volumes
2021 Tank et al. [119]	UK Biobank (*n* = 32,790)	*APOE*, summary statistics	Univariate imaging—Multivariate genetic	PGS-based association	Volumes of total GM, WM, WMH, whole brain, left/ right hippocampus	Left hippocampus	LOAD-PGR was associated with smaller HV and aspects of cognitive ability in healthy adults and could supplement *APOE* status in risk stratification of cognitive impairment/LOAD
**Pathophysiological pathway: Neurodegeneration (FDG PET)**							
2010 Corneveaux et al. [120]	Multiple cohort (*n* = 1728)	*KIBRA* rs17070145	Univariate imaging—Univariate genetic	Candidate-based association	Glucose metabolism	Entorhinal cortex, hippocampus, middle temporal gyrus, posterior cingulate cortex, superior frontal gyrus, primary visual cortex	Non-carriers of the *KIBRA* rs17070145-T had increased risk of LOAD in an association study of 702 neuropathologically verified expired subjects and in a combined analysis of 1026 additional living and expired subjects
2014 Lehmann et al. [77]	52 AD	*APOE*	Multivariate imaging—Univariate genetic	Candidate-based association	PIB DVR, FDG SUVR	Lateral temporoparietal cortex, precuneus, posterior cingulate cortex, middle frontal gyrus, etc	*APOE * ε4+ AD patients showed lower global amyloid burden and greater medial temporal hypometabolism compared with matched *APOE * ε4- patients
2018 Miller et al. [121]	ADNI (*n* = 695)	*EXOC3L4*	Univariate imaging—Multivariate genetic	WGS	Global cortical glucose metabolism	Whole brain cortex	*EXOC3L4* gene, was identified as significantly associated with global cortical glucose metabolism. Three loci that may affect splicing within *EXOC3L4* helped to the association
2018 Kong et al. [122]	ADNI (37 HC, 59 AD, 126 MCI)	Genome-wide genotyping	Univariate imaging—Univariate genetic	GWAS	ROI glucose metabolic uptake	Left and right angular, temporal gyri, bilateral posterior cingulate	A genome-wide significant SNP rs12444565 in the *RBFOX1*, four suggestive loci (rs235141, rs79037, rs12526331 and rs12529764) were associated with ^18^F-FDG
2020 Seo et al. [123]	KBASE (336 HC, 84 AD, 136 MCI)	132 AD candidate genes	Multivariate imaging—Multivariate genetic	Candidate-based association	Aβ deposition, region cerebral glucose metabolism/ cortical thickness, HV	AD-signature cortical, hippocampus	Several novel loci for common variants were associated with AD pathology (*PIWIL1*, *NME8* and *PSEN2*, *PSEN1*, *CASS4*). Cases carrying rare variants in *LPL*, *FERMT2*, *NFAT5*, *DSG2*, and *ITPR1* displayed associations with the neuroimaging features
2021 Wang et al. [124]	ADNI (*n* = 586)	Genome- wide genotyping	Univariate imaging—Multivariate genetic	GWAS	Glucose metabolic uptake in ROIs	Left angular gyri, bilateral posterior cingulate gyrus, right /left middle/inferior temporal gyrus	Two genome-wide significant SNPs (rs4819351, rs13387360) in *AGPAT3* and *LOC101928196* served as protective sites to regulate the decline of glucose metabolism
2019 Li et al. [80]	ADNI (37 HC, 86 AD, 188 MCI)	Genome-wide genotyping	Univariate imaging—Multivariate genetic	GWAS	Glucose metabolic uptake in ROIs	Frontal, lateral parietal, lateral temporal regions, anterior/posterior cingulate regions	Indirect genetic effects on certain chemical compound or protein translocation were reflected in the PET scans and may be associated with AD
**Pathophysiological pathway: Neurodegeneration (fMRI)**							
2000 Bookheimer et al. [125]	30 HC	*APOE*	Univariate imaging—Univariate genetic	Candidate-based association	Patterns of brain activation	Left hippocampal, parietal, prefrontal cortices	Both the magnitude and the extent of brain activation during memory-activation tasks in regions of the left hippocampal, parietal, and prefrontal regions, were greater among the carriers of the *APOE * ε4 allele than among the carriers of the *APOE ɛ3* allele
2011 Erk et al. [126]	109 HC	*CLU* rs11136000	Univariate imaging—Univariate genetic	Candidate-based association	FC	Hippocampus, prefrontal cortex	Healthy carriers of the variant exhibited altered coupling between hippocampus and prefrontal cortex during memory processing
2011 Lancaster et al. [127]	43 HC	*CLU* rs11136000	Univariate imaging—Univariate genetic	Candidate-based association	Working memory values based on brain activity	Frontal, posterior cingulate cortex, hippocampus	Participants with the *CLU* risk genotype had higher activity than participants with the protective allele in frontal and posterior cingulate cortex and hippocampus
2014 Green et al. [128]	131 HC	*APOE*, *CLU*	Univariate imaging—Multivariate genetic	Candidate-based association	ROI BOLD signal change	Hippocampus, MTL	*APOE* ε4 and *CLU*-C had an additive effect on brain activity, that is, increased combined genetic risk was associated with decreased brain activity during executive attention, including in the MTL
2014 Guerini et al. [129]	*n* = 1680	*SNAP-25* SNP	Univariate imaging—Univariate genetic	Candidate-based association	FMRI task accuracy	Cingulate cortex, frontal, temporoparietal cortices	FMRI analyses indicated that *SNAP-25* genotypes correlated with a significantly decreased brain activity in the cingulate cortex and in the frontal (middle, superior gyri) and the temporo-parietal (angular gyrus) area
2014 Liu et al. [130]	Han Chinese (21 HC, 46 MCI)	*TOMM40* rs157581	Univariate imaging—Univariate genetic	Candidate-based association	ALFF	Bilateral superior frontal gyrus, bilateral lingual gyrus, right calcarine sulcus, left cerebellar	*TOMM40* rs157581 polymorphism may modulate regional spontaneous brain activity and relate to the progression of aMCI
2015 Lancaster et al. [131]	85 HC	*CLU* rs11136000	Multivariate imaging—Univariate genetic	Candidate-based association	Working memory task accuracy, GM density	Hippocampus, prefrontal, limbic areas	Young individuals with the *CLU* rs11136000-C had higher activation levels in prefrontal and limbic areas during a working memory task. And there were subtle reductions in GM in the right hippocampal formation in carriers of the risk variant
2015 Zhang et al. [132]	360 HC	*BIN1* rs744373	Multivariate imaging—Univariate genetic	Candidate-based association	Working memory, GM volume, FC	Whole brain	Healthy homozygous carriers of the rs744373 risk allele exhibited worse high-load working memory performance, larger HV and lower FC between the bilateral hippocampus and right dorsolateral prefrontal cortex
2017 Sun et al. [133]	32 HC, 32 MCI	*PICALM* rs3851179	Univariate imaging—Univariate genetic	Candidate-based association	FC	DMN	The *PICALM* rs3851179 polymorphism significantly affected the DMN network in MCI
2017 Xiao et al. [110]	*n* = 231	*APOE*, summary statistics	Univariate imaging—Multivariate genetic	PGS-based association	Activation in hippocampus ROI	Hippocampus	There was a cumulative deleterious effect of LOAD risk genes on hippocampal function even in healthy volunteers
2017 Su et al. [134]	131 HC, 87 MCI	*APOE*, summary statistics	Univariate imaging—Multivariate genetic	PGS-based association	FC in ROIs of DMN	Temporal cortex	The pMCIs exhibited tremendous decrements in DMN connections that were partially determined by the AD-related risk alleles
2018 Korthauer et al. [135]	76 HC	*APOE*	Multivariate imaging—Univariate genetic	Candidate-based association	Graph analysis of network efficiency	Whole brain functional-structural network	ε4 carriers had significantly lower global and local efficiency of the integrated resting-state structural connectome compared to non-carriers
2021 Franzmeier et al. [136]	Multiple cohort (*n* = 378)	*BDNF*_*Val66Met*_ SNP	Univariate imaging—Univariate genetic	Candidate-based association	FC	DMN, DAN, SAL, CON	*BDNF*_*Val66Met*_ was associated with a higher vulnerability of hippocampus-frontal connectivity to primary AD pathology
2019 Chandler et al. [137]	*n* = 75	*APOE*, summary statistics	Univariate imaging—Multivariate genetic	PGS-based association	Whole-brain gmCBF	Frontal cortex	The results found a reduction in gmCBF in *APOE * ε4 carriers, a negative relationship between AD-PGS and gmCBF, and regional reductions in gmCBF in individuals with higher AD-PGS across the frontal cortex
2019 Axelrud et al. [138]	Multiple cohorts (*n* = 636)	*APOE*, summary statistics	Univariate imaging—Multivariate genetic	PGS-based association	FC among main nodes for 10 tau pathology networks	Precuneus, superior temporal gyrus	The PGS was associated with the connectivity between the right precuneus and the right superior temporal gyrus
2020 Chandler et al. [139]	*n* = 608	*APOE*, summary statistics	Univariate imaging—Multivariate genetic	PGS-based association	Bilateral hippocampus bold parameters	Hippocampus	AD-PGS, not *APOE*, selectively influenced activity within the HC in response to scenes, while other perceptual nodes remained intact
**Pathophysiological pathway: Neurodegeneration (DTI)**							
2010 Smith et al. [140]	23 HC, 42 AD	*APOE*	Univariate imaging—Univariate genetic	Candidate-based association	FA	Inferior temporal lobe, amygdala/ hippocampal head region	Reduced FA was observed in the fronto-occipital and inferior temporal fasciculi (particularly posteriorly), the splenium of the corpus callosum, subcallosal white matter and the cingulum bundle
2005 Nierenberg et al. [141]	29 HC	*APOE*	Univariate imaging—Univariate genetic	Candidate-based association	FA, axD, radD	Parahippocampal region	The *APOE * ε4 carriers showed significantly lower fractional anisotropy and higher radial diffusivity in the parahippocampal WM 15 mm below the anterior commissure-posterior commissure plane than noncarriers
2014 Warstadt et al. [142]	*n* = 481	Genome-wide genotyping	multivariate imaging—multivariate genetic	GWAS	Diffusion tensor, FA	Corpus callosum, fornix, internal capsule, inferior fronto-occipital fasciculus	A follow-up analysis detected WM associations with rs5882 in the opposite direction
2015 Liang et al. [143]	126 HC	*SORL1* rs2070045	Univariate imaging—Univariate genetic	Candidate-based association	FA, MD, axD, radD	Bilateral cingulum, cingulum hippocampal area	Sex moderated the effects of the *SOR1* gene rs2070045 polymorphism on cognitive impairment and disruption of the cingulum hippocampal integrity in healthy elderly
2016 Foley et al. [106]	*n* = 197	*APOE*, summary statistics	Multivariate imaging—Multivariat genetic	PGS-based association	FA	Right cingulum	Fractional anisotropy of the right cingulum was inversely correlated with AD polygenic risk scores
2017 Cavedo et al. [144]	74 HC	*APOE*	Univariate imaging—Univariate genetic	Candidate-based association	FA, MD, radD, axD	Cingulum, corpus callosum, inferior fronto-occipital, inferior longitudinal fasciculi, internal, external capsule	These findings indicated a modulatory role of *APOE * ε4 on WM microstructure in elderly individuals at risk for AD suggesting early vulnerability and/or reduced resilience of WM tracts involved in AD
2018 Rutten-Jacobs et al. [145]	UK Biobank (*n* = 8448)	Genome-wide genotyping	Univariate imaging—Multivariate genetic	GWAS	FA, MD, WMHV	White matter hyperintensity	A novel genome-wide significant locus *VCAN* rs13164785 on chr5q14 was identified, which may work in the mechanisms underlying microstructural integrity of the WM measured as FA and MD
2019 Gu et al. [146]	GWAS Summary Statistics	*PSEN1*	Multivariate imaging—Univariate genetic	Meta- analysis	WM integrity, cerebral amyloid deposition and brain metabolism	Whole brain	*PSEN1* mutation associated with WM changes and amyloid deposition occurred in AD. Increased MD was observed and showed significant increase with amyloid deposition
2020 Yan et al. [147]	ADNI (34 HC, 36 AD, 49 MCI)	34 GWAS AD risk SNPs	Univariate imaging—Multivariate genetic	Candidate-based association	Fibre anisotropy, fibre length and density	278 brain ROIs	Rs10498633 in *SLC24A4* was found to be significantly associated with anisotropy, total number and length of fibres. *APOE* rs429358 showed nominal significance of association with the density of fibres between subcortical and cerebellum regions
2020 Horgusluoglu-Moloch et al. [148]	ADNI (34 HC, 15 AD, 56 MCI)	23 AD genes	Univariate imaging—Multivariate genetic	Candidate-based association	FA, MD, radD, axD, LIN, SPH, PLA, MOD	Hippocampus, cingulum, parahippocampal gyrus right, sagittal stratum, etc	A SNP rs2203712 in *CELF1* was most significantly associated with several DTI-derived features in the hippocampus, the top ranked brain region

*ALFF* amplitude of low-frequency fluctuations, *axD* axial diffusivity, *CVR* rating of cerebrovascular disease, *DAN* dual attention network, DMN default mode network, *DVR* distribution volume ratios, *FA* fractional anisotropy, *FC* functional connectivity, *FN* frontoparietal network, *HV* hippocampal volume, *ICV* intracranial volume, *gmCBF* grey-matter cerebral blood flow, *KBASE* Korean brain aging study for early diagnosis and prediction of Alzheimer’s disease, *KL-VS*^*he*t^ KL-VS heterozygosity, *LIN* linearity of the tensor, *MD* mean diffusivity, *MOD* mode of the tensor, *MTA* medial temporal atrophy, *MTL* medial temporal lobe, *PLA* planarity of the tensor, *pMCI* progressive MCI, *radD* radial diffusivity, *SMC* significant memory concern, *SN* salience network, *SPH* sphericity of the tensor, *SUVR* standard update value ratios, *TBV* total brain volume, *WMH* white matter hyperintensity

#### Imaging genomics analysis of “A” biomarker

Of the ATN framework, “A” refers to the Aβ plaque biomarker, including cortical amyloid PET ligand binding and CSF Aβ_42_ level. The deposition of amyloid plaques in the brain is one of the two main pathological signs of AD. As a reliable imaging phenotype of AD, amyloid PET can selectively detect Aβ deposition in the brain. A number of studies using amyloid PET have investigated how various genetic variants influence Aβ burden.

At the candidate-gene level, Drzezga et al. [70] examined the effect of *APOE* genotype on the levels of [^11^C] PiB PET Aβ plaques in AD patients using the VBM-based method and regression analysis. The results showed higher levels of Aβ plaque deposition in ε4-positive patients in bilateral temporoparietal and frontal cortical areas. Apostolova et al. [17] investigated the associations of the top 20 AD risk variants with brain amyloidosis using ADNI datasets by multivariable linear regression analysis. The results showed that the *ABCA7* gene has the strongest association with amyloid deposition, while the *APOE * ε4 and *FERMT2* genes show stage-dependent associations with amyloid deposition, especially in the MCI stage.

At the genome-wide level, Yan et al. [149] conducted a GWAS meta-analysis using [^11^C] PiB PET imaging from the ADNI datasets, and found that the *APOE* region showed the most significant association with brain Aβ burden. Ramanan et al. [150] performed the first GWAS of cortical Aβ burden in humans using data from ADNI-2 and ADNI-Grand Opportunity and reported that *APOE* and *BCHE* (*BUCHE*) are independent regulators of amyloid deposition in the brain, accounting for nearly 15% of the variance in cross-sectional amyloid load. At the polygenic level, Tan et al. [151] observed a strong association between polygenic hazard scores and Aβ uptake. A detailed summary of these findings is shown in Table 3.

#### Imaging genomics analysis of “T” biomarker

“T” refers to the tau biomarker, including CSF phosphorylated tau and cortical tau PET. The twisted strands of the protein tau (tangles) inside neurons are the other pathological marker of AD. Although tau pathology serves as a primary brain pathology associated with cognitive impairment in AD, most previous studies have focused on CSF tau levels, which reflect tau production rather than the amount of pathological tau deposition in the brain. The recent advent of AV1451 tau-PET imaging has allowed the assessment of fibrillary tangles in the living brain.

At the candidate-gene level, Smith et al. [83] reported that the [^18^F] AV1451 tau-PET imaging is strongly correlated with tau neuropathology in *MAPT* (microtubule-associated protein tau) mutation carriers. After that, Yan et al. [88] explored the association of sex and *APOE * ε4 with brain tau deposition and atrophy in older adults with AD, and found that female *APOE * ε4 carriers (FACs) have elevated tau-PET SUVR in comparison to non-FACs. Therriault et al. [86] and Neitzel et al. [89] independently evaluated different datasets and reported that *APOE * ε4 is associated with higher tau accumulation and that this association is independent of amyloid burden. Regarding other AD candidate genes, Franzmeier et al. [87, 90] and Neitzel et al. [91] suggested that the *BIN1* rs744373 SNP and Klotho-VS heterozygosity are associated with higher and lower pathologic tau levels, respectively, by analyses of variance and multiple linear regression.

At the genome-wide level, Ramanan et al*.* [152] conducted the first neuroimaging GWAS of tau pathology in 754 individuals. The findings not only confirmed the association of *MAPT* with tau burden, but also identified the *NTNG2*-rs75546066 locus as having a novel protective effect against tau pathology.

At the polygenic level, Sun et al. [92] assessed PGS values as a predictor of tau pathology in non-demented individuals. The results showed that higher PGS values were correlated with elevated tau-PET uptake values, and the significance remained when *APOE* was regressed.

#### Imaging genomics analysis of “N” biomarker

“N” refers to neurodegeneration or neuronal injury, including CSF total tau level, [^18^F]FDG PET hypometabolism, and atrophy on sMRI. Among them, sMRI is the most widely used technology in imaging biomarker genomics studies to extract targeted imaging phenotypes, with increased discriminative power and improved biological interpretability. [^18^F]FDG PET can detect brain glucose metabolism and provide important pathological staging information. Several studies have also investigated how various genetic variants influence brain glucose metabolism.

At the candidate-gene level, the associations of *APOE* with MRI genotypes have been investigated, especially between ε4 carriers and noncarriers. For example, Wolk et al. [95] found that the *APOE* genotype affects cognitive and anatomic phenotypic expression of AD, in that the ɛ4 carriers with mild AD show greater impairment on measures of memory retention and greater MTL atrophy compared to noncarriers who are more impaired in working memory and show greater frontoparietal atrophy. Risacher et al. [153] found that the annual percent change rate of MRI atrophy is influenced by the *APOE* genotype. Morgen et al. [99] found that the genetic interaction between *PICALM* and *APOE* is associated with brain atrophy and cognitive impairment using univariate analysis of variance. Moreover, Biffi et al. [96] investigated the impact of multiple GWAS-validated and GWAS-promising candidate loci on hippocampal volume, amygdala volume, WM lesion volume, entorhinal cortical thickness, parahippocampal gyrus thickness and temporal pole cortical thickness. The study indicated that genetic variants that modulate AD risk as revealed in previous GWASs may influence neuroimaging measures. In addition, *BIN1* and *CNTN5* were identified as two novel loci that show associations with multiple MRI characteristics, which are of interest for further studies. Regarding brain glucose metabolism biomarkers, Lehmann et al. [77] assessed the relationships between glucose metabolism and *APOE* genotype in clinical AD patients, with one-way analysis of variance and Tukey’s *post-hoc* test, and found a greater degree of medial temporal hypometabolism in *APOE * ε4 carriers. Miller et al. [121] explored and confirmed the associations between rare variants in splicing regulatory element loci of *EXOC3L4* and global cortical glucose metabolism in the ADNI cohort. Notably, Seo et al. [123] analyzed the effects of 132 selected susceptibility genes previously identified to be associated with LOAD, on neurodegenerative brain features by using neuroimaging data from the KBASE (Korean Brain Aging Study for Early Diagnosis and Prediction of Alzheimer’s disease) cohort, including [^11^C]PiB PET, [^18^F]FDG PET, and MRI. In contrast to previous studies, this study utilized five in vivo AD pathologies and associated them with both common and rare genetic variants by performing targeted sequencing of 132 candidate genes.

At the genome-wide level, Kong et al. [122] performed the first GWAS examining brain FDG metabolism in 222 subjects from the ADNI cohort in 2018, and identified *RBFOX1* (RNA-binding Fox1) SNP rs12444565 to have a strong association with brain glucose metabolism. Wang et al. [124] identified two genome-wide significant SNPs, rs4819351 in *AGPAT3* (1-acylglycerol-3-phosphate O-acyltransferase 3) and rs13387360 in *LOC101928196*, that had strong protective effects against the longitudinal metabolic decline in the right temporal gyrus and the left angular gyrus, respectively. At the polygenic level, Desikan et al. [102] reported that the polygenic hazard score was associated with longitudinal MRI-derived volume loss in the entorhinal cortex and hippocampus.

In addition to the above “N” biomarker, many other advanced MRI technologies have also been applied to study the influence of genetic variation on functional or WM alterations. Based on the DTI technology, WM alterations have been found in AD and MCI, and *APOE* may play a role in modulating these alterations [140, 141, 143, 144, 146–148]. Some researchers have reported differences in WM integrity between healthy APOE ɛ4 carriers and noncarriers by using diffusion parameters, including fractional anisotropy, mean diffusivity, and radial diffusivity. In addition, Gu et al. [146] performed a meta-analysis of associations of the *PSEN1* genotype with WM integrity and brain metabolism, and indicated that *PSEN1* is associated with mean diffusivity increase in DTI markers and decreased brain metabolism. Foley et al. [106] analyzed associations between AD polygenic risk scores and diffusion-weighted parameters in young adults, and revealed that the fractional anisotropy of the right cingulum is correlated with AD polygenic risk score. Regarding fMRI, both resting-state fMRI and task-fMRI were conducted to evaluate associations of brain activity with *APOE* and other AD risk genes [129, 130, 133, 136]. Many of these studies were performed in healthy older adults [125–128, 131, 132, 135] to investigate potential risk-allele influences on functional brain activity. It is worth noting that Jahanshad et al. [98] explored the heritability of various brain connections based on genome-wide associations and discovered the *SPON1* (F-spondin) rs2618516 variant to affect dementia severity. Besides, Su et al. [134] investigated the associations between AD PGS and functional connectivity in the default mode network, and found significant correlations in the temporal cortex.

**Fig. 5:**
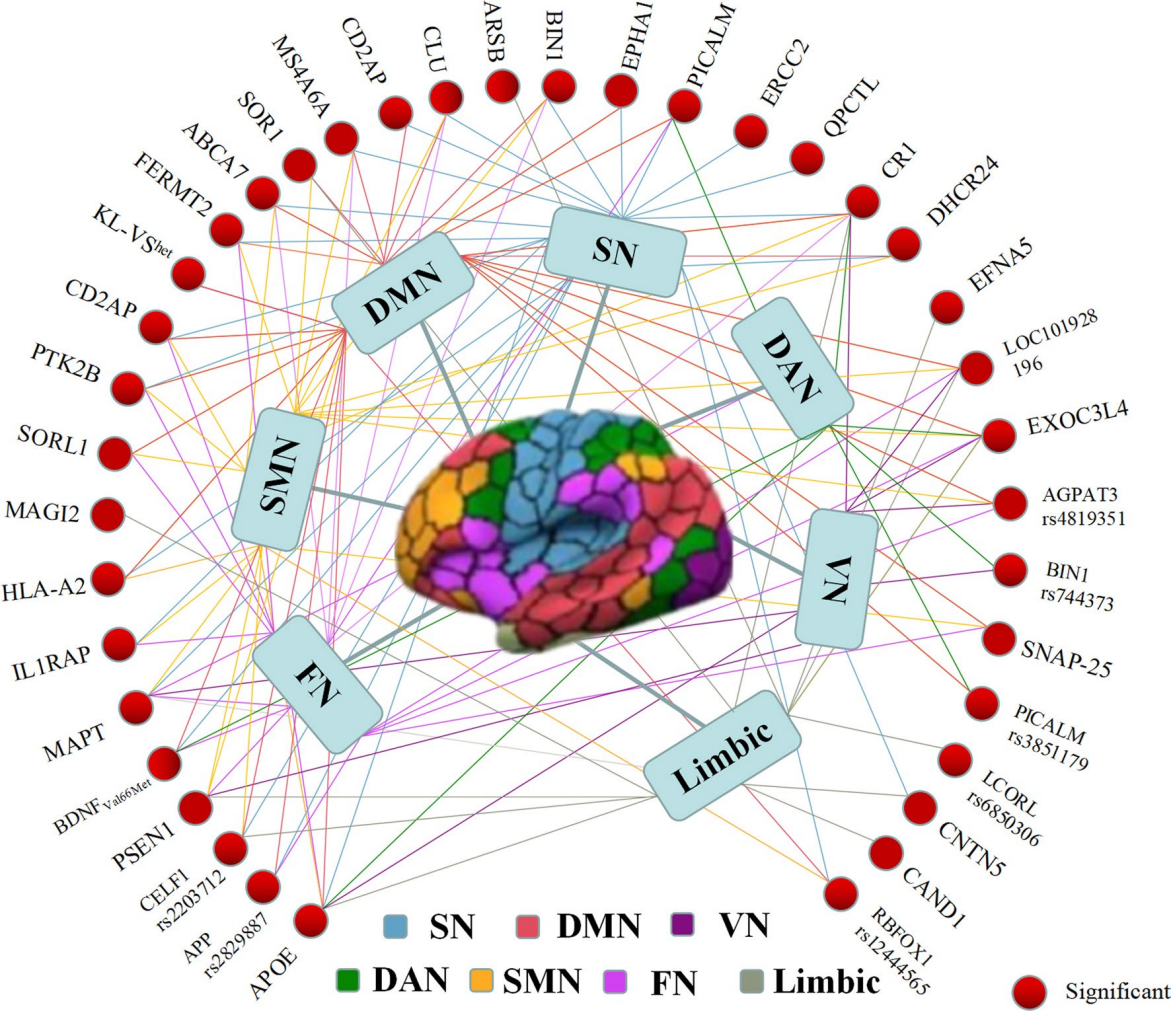
The relationship between genomic data and 7 specific brain networks from Yeo’s template. These associations are respectively marked in colors consistent with the corresponding brain networks. *DAN* dual attention network, *DMN* default mode network, *FN* frontoparietal network, *SMN* somatomotor network, *SN* salience network, *VN* visual network

Figure 5 illustrates the mapping of associations between genomic data and brain functional networks, which are classified into 7 brain networks according to Yeo’s template, including visual network, somatomotor network, dual attention network, salience network, limbic network, frontoparietal network, and default mode network. In summary, associative studies of AD brain imaging biomarker genomics can provide new insights into the pathological and genetic mechanisms underlying AD. In addition, the number of genome-wide studies is relatively small compared with candidate-gene association studies, which may be caused by the scarcity of neuroimaging data. However, studies only focused on selected candidate genes may ignore potential interactions among multiple significant genetic variants, which emphasizes the necessity of genome-wide interaction and PGS analyses with improvement in multimodal imaging databases.

### AD diagnosis and prognosis based on brain imaging biomarker genomics

Recent advances of artificial intelligence (AI) techniques enable automatic combination of multimodal neuroimaging and genomics data to provide complementary and comprehensive information for AD diagnosis and prognosis. Specifically, ML methods have been widely implemented in computer-aided diagnosis of AD, including traditional ML models and advanced DL algorithms. The traditional classification models include support vector machine (SVM), random forest (RF), linear discriminant analysis (LDA) and regression models (RL). De Velasco et al. [154] compared performances of ML models least absolute shrinkage and selection operator (LASSO), k-nearest neighbour (KNN), and SVM in predicting LOAD from genetic variation data, with SVM showing the best performance (AUC = 0.72). In addition, *APOE* genotype is the most commonly utilized genomic data. For example, Gray et al. [155] performed multi-modality classification based on joint embedding of sMRI, FDG PET, CSF biomarkers, and *APOE* genotype data, using a multimodal RF model and a fourfold cross validation (CV) to predict AD, and achieved an accuracy of 89% in classifying AD from healthy controls. Similarly, by combining sMRI, FDG PET, CSF biomarkers, *APOE* genotype, age, sex and body mass index, Kohannim et al. [156] selected a SVM model and performed leave-one-out CV for AD and MCI classification and prediction of future cognitive decline within 1 year, and achieved a maximum of 90% accuracy for AD vs healthy controls. To distinguish between stable and progressive MCI, Dukart et al. [157] used a plain Bayesian (naive Bayesian, NB) algorithm based on *APOE* genotype, neuropsychological assessment, sMRI, and FDG PET, achieving an accuracy of approximately 87%. Moreover, Bi et al*.* [158] combined fMRI and SNP data and used the multimodal RF algorithm to distinguish AD from normal control, and finally obtained AD prediction accuracy of 87%. Varol et al. [159] proposed the heterogeneity through discriminative analysis (HYDRA) algorithm to predict AD based on combined sMRI and SNP data, with the highest AUC value being 0.942.

On the other hand, in the context of DL method, Liu et al. [160] integrated DTI and SNP data with deep convolutional neural networks for prediction of AD, and obtained AUC values of 0.8571, 0.8291, 0.8583, and 0.7756 at baseline, 6 months, 12 months and 24 months, respectively. Similarly, combining sMRI and SNP data, Ning et al. [68] used a neural network to predict AD and achieved an AUC value of 0.992. Moreover, based on sMRI, demographics, neuropsychological assessment and *APOE* genotype data, Spasov et al. [161] used the convolutional neural network model to distinguish MCI patients who would develop AD within 3 years from patients with stable MCI, with an AUC value of 0.925. By combining sMRI, FDG PET and SNP data, Zhou et al. [162] conducted three-stage deep feature learning and fusion to simultaneously predict HC, MCI and AD, with an accuracy of 65%, which was higher than that of other ML classification methods. In addition to the joint use of imaging and clinical information, combination with multiomics information is also an emerging trend in AD research. Shigemizu et al. [163] integrated genomic data and microRNA expression profiles to construct a proportional hazards model-based prognostic model to identify MCI individuals at high risk of AD. A consistency index of 0.702 was obtained on an independent test set. A detailed list of machine learning-based studies of imaging biomarker genomics is provided in Table 4.

**Table 4:** Application of machine learning based on imaging biomarker genomics in AD diagnosis and prognosis

Method	Year	Modality	Model	Dataset	CV	Neural location	Results
Machine learning	2010 [156]	sMRI, FDG PET, CSF, *APOE* genotype, age, sex, body mass index	SVM	HC: 213 AD: 158 MCI: 264	LOOCV	Hippocampal, ventricular, temporal lobe	A maximum up to 90% accuracy for AD
	2013 [155]	sMRI, FDG PET, CSF, *APOE* genotype	MRF	HC: 35 AD: 37 MCI: 75	Fourfold CV	Whole brain	An accuracy of 89% for AD
	2014 [164]	sMRI, FDG PET, CSF, SNP	SVM	HC: 47 AD: 49 MCI: 93	Tenfold CV	Whole brain	An accuracy of 71% among HC, MCI and AD
	2016 [157]	*APOE* genotype, neuropsychological assessment, sMRI, FDG PET	NB	HC: 112 AD: 144 sMCI: 265 pMCI: 177	independent test set	Whole brain	An accuracy of 87% in identifying pMCI from sMCI
	2017 [159]	sMRI, SNP	HYDRA	HC: 139 AD: 103	–	Hippocampus, entorhinal cortex frontal lobe	The highest AUC value of 0.942 for AD
	2017 [165]	sMRI, SNP	SVM	HC: 204 AD: 171 MCI: 362	Tenfold CV	Whole brain	An accuracy of 80.8% identifying pMCI from sMCI
	2019 [158]	fMRI, SNP	MRF	HC: 35 AD: 37	–	Olfactory cortex, insula, posterior cingulate gyrus and lingual gyrus	An accuracy of 87% AD prediction
	2019 [154]	SNP	LASSO, KNN, SVM	HC: 371 AD: 267	CV	–	The highest reached 0.72 of the AUC
	2019 [166]	*APOE*, PET, PGS	LR	HC: 224 AD: 174 MCI: 344	–	Whole brain	An AUC value of 0.69 using PGS and *APOE* to predict amyloid state
	2020 [167]	sMRI, FDG PET, AV45 PET, DTI, resting-state fMRI, *APOE* genotype	MKL	HC: 35 AD: 33 sMCI: 30 pMCI: 31	LOOCV	Whole brain	An accuracy of 96.9% in identifying pMCI from sMCI
Deep learning	2017 [162]	SNP, sMRI FDG PET	DFFF	HC: 226 AD: 190 MCI: 389	Twentyfold CV	Whole brain	An accuracy of 0.65 among HC, MCI and AD
	2018 [68]	sMRI, SNP	NN	HC: 225 AD: 138 MCI: 358	Fivefold CV	16 ROIs (hippocampus, entorhinal cortex, parahippocampal gyrus, amygdala, precuneus, etc.)	An AUC value of 0.992 using combined features
	2019 [161]	sMRI, demographic, neuropsychological assessment, *APOE* genotype data	CNN	HC: 184 AD: 192 sMCI: 228 pMCI: 181	Tenfold CV	Whole brain	An AUC value of 0.925 for pMCI prediction
	2019 [160]	DTI, SNP	DCNN	HC: 100 AD: 51	Fivefold CV	Temporal lobes (including the hippocampus) and the ventricular system	The highest AUC value of 0.858
	2021 [61]	MRI, SNP, electronic health records	CNN	ADNI	independent test set	Whole brain	A maximum up to 87% accuracy

*CNN* convolutional neural network, *CV* cross validation, *DCNN* deep CNN, *DFFF* deep feature learning and fusion framework, *HYDRA* heterogeneity through discriminative analysis, *LOOCV* leave-one-out CV, *MKL* multiple kernel learning, *MRF* multimodal random forest, *NN* neural network, *pMCI* progressive MCI, *sMCI* stable MCI

In summary, the above-mentioned studies show that ML methods with multimodal data such as imaging, clinical and multiomics data as input measures, are valuable tools for prognosis and risk stratification of AD with improved accuracy.

## Key considerations and perspectives regarding AD imaging biomarker genomics

As a novel approach, the brain imaging biomarker genomics technique still needs further optimization, mainly in the following aspects.

### Variable control in calculations

Calculations in AD imaging biomarker genomics can be influenced by various factors. Differences in physiological, demographic, and environmental factors can affect heritability estimates and measurements of brain-related features, which may obscure the disease-related effects and limit the utility of brain-related features as endophenotypes. Some recent studies have investigated associations of *APOE* ε4 status and sex  with cognitive memory [88, 95, 168–170]. Therefore, these potential confounding factors should be included as covariates to improve comparability and reliability of findings. In particular, sex, education and* APOE* ε4 status are always used as covariates in large imaging–genomics GWAS and meta-analyses. Another way to avoid these potential influences is to carry out studies in healthy individuals or in a single ethnic or sex group. Ethnicity is another critical factor. Independent replication and meta-analyses remain the most reliable methods for reducing false-positive findings [171]. Comprehensive and ethnicity-homogeneous databases are needed to verify the generalizability and robustness of significant results. Compared to candidate-gene analyses which could not account for epistatic effects between genes, genome-wide analysis is more unbiased, thus underscoring again the significance of large samples in the future.

### Use of prior knowledge  on calculations

Interpretation of results is a focus of brain imaging biomarker genomics for AD. The use of prior knowledge, such as the Allen Human Brain atlas (AHBA), can facilitate calculations in brain imaging biomarker genomics and correlate spatial variations at the molecular scale with macroscopic neuroimaging phenotypes. For example, Franzmeier et al. [90] and Neitzel et al. [91] have used the AHBA to explore associations of *BIN1* rs744373 and KL-VS heterozygosity with tau accumulation, respectively. Moreover, Sepulcre et al. [172] have developed a novel graph theory approach named directional graph theory regression (DGTR) to investigate the intersection of tau/Aβ pathological changes in the brain and the genetic transcriptome of AHBA. This approach can potentially be applied to explore more phenotype-genotype associations. Taken together, increasing the sensitivity and power of genetic effects, adequately utilizing ROIs, reliably stimulating responses, and highlighting differences among individuals are extremely necessary. For example, identifying differential masks first, as ROIs on a unique dataset, will lead to higher sensitivity.

### Generalization of multivariate approaches beyond GWAS

Currently, biomarkers derived from GWASs were usually identified based on clinical outcomes. This approach has both advantages and disadvantages. Compared with imaging phenotypes limited by the scarcity of neuroimaging data, it is easier and more feasible to obtain a large number of clinical phenotypes, thus better meeting the prerequisites of large-scale GWAS and reducing greatly false-positive results. However, the accuracy of this approach is influenced by the sample size and statistical methods. In contrast, combining neuroimaging markers with GWAS genetic phenotypes can explain potential biological mechanisms in relatively small sample sizes.

Therefore, imaging biomarker genomics studies are gaining novel insights in comparison to traditional GWAS analyses. For example, data-driven multivariate approaches are emerging to explain more imaging-genetic variants, such as sparse canonical correlation analysis and parallel independent component analysis [69]. These multivariate approaches have provided increased detection power and put forward new technical challenges, including data dimensionality reduction and feature selection strategies. Besides, the GWAS analysis pipelines are also expected to be further optimized to process complex and high-dimension genetic data automatically.

### Combination of AI and brain imaging biomarker genomics

Currently, ML methods have been widely used for AD diagnosis and prognosis. On the one hand, traditional ML and advanced DL algorithms are relatively mature computational methods in AD imaging studies and include model building, feature processing and model evaluation. On the other hand, combination of genomics calculations with ML algorithms has not been widely performed. Applications of deep neural networks in genetic studies are still scarce, although seminal studies have demonstrated the accessibility of deep neural networks to DNA sequencing data, resulting in generation of DeepBind, DeepSEA and Basset networks [173–176]. Therefore, more efforts should be focused on the development of solutions for technical challenges especially for DL algorithms, such as how to reduce dimensionality of multimodal data, how to integrate imaging and genomics data, and how to interpret the effectiveness of DL features.

### Integration of multiomics data

AD imaging biomarker genomics research has identified numerous novel genetic variants and gained insights into disease mechanisms. However, the pathological mechanisms underlying AD are still far from well understood. Apart from the development of methods, the integration of multimodal imaging data and genomics, microRNAomics, metabolomics, proteomics, and transcriptomics will continue to be an important research direction. Genomics is now the most mature omic technology with development of high-throughput genotyping arrays and sequencing strategies. Other omic technologies have also been incorporated into research domains. For example, mass spectrometry-based proteomics has driven deep profiling of the proteome in AD. The AD proteomic review by Bai et al. [177] indicated that proteomics-driven systems biology would be a promising frontier to link genotype, proteotype, and phenotype and accelerate improvement in AD models and treatment strategies. Besides, neuroimaging markers are not limited to MRI and PET markers. During the last few decades, EEG and MEG techniques have also been commonly applied in AD studies. For instance, alterations of brain rhythms and functional connectivity have been revealed in EEG and MEG studies [178–180]. Relationships between various AD genetic risk factors and EEG phenotypes have also been reported [181–184]. Hence, compared with a single omics category, integration of multiomics information allows systemic exploration at multiscale layers to better understand the comprehensive biological information flow that underlies the disease and to pave the way for precision medicine.

## Conclusions

The field of brain imaging biomarker genomics has made tremendous progress in the last decade to capture novel genetic variants and explore potential disease pathophysiology mechanisms. Future studies in this field are anticipated to move forward to precise medicine, to identify significant findings that can be used in clinical practice, and to achieve computer-aided AD diagnosis and prognosis. Therefore, further development of current research methods and integration of information will continue to be an important research direction. There is no doubt that unbiased genome-wide approaches remain critical, and replication studies are necessary. Advances in next-generation sequencing approaches coupled with more refined brain mapping (such as AHBA that maps genetic variants to brain tissues) are increasingly promoting the interpretability of findings from imaging biomarker gemonics. In addition, DL algorithms allow for integration of multiple preprocessing steps into a single model to improve AD diagnosis and prognosis. In summary, current studies in the AD imaging biomarker genomics field have profiled the brain mechanisms at an unprecedented scale, raising new hypotheses for subsequent validation.

## Supplementary Information

**Additional file 1.** Search strategy for literature.

## Data Availability

Not applicable.

## Abbreviations

AD: Alzheimer’s disease
ADNI: Alzheimer’s disease neuroimaging initiative
AHBA: Allen human brain atlas
AI: Artificial intelligence
*APOE*: Apolipoprotein E
*APP*: Amyloid precursor protein
AUC: Area under curve
Aβ: Amyloid β
CSF: Cerebrospinal fluid
CV: Cross validation
DGTR: Directional graph theory regression
DL: Deep learning
DPABI: Data processing and analysis for brain imaging
DPARSF: Data processing assistant for resting-state fMRI
DTI: Diffusion tensor imaging
EEG: Electroencephalography
FAC: Female *APOE* ɛ4 carriers
FDG: Fluorodeoxyglucose
fMRI: Functional MRI
FSL: FMRIB’s Software Library
GM: Grey matter
GWAS: Genome-wide association analysis
GWAX: Genome-wide association study by proxy
HC: Healthy controls
HYDRA: Heterogeneity through discriminative analysis
IGAP: International genomics of Alzheimer’s project
KBASE: Korean brain aging study for early diagnosis and prediction of Alzheimer’s disease
KNN: K-nearest neighbour
LASSO: Least absolute shrinkage and selection operator
LDA: Linear discriminant analysis
LOAD: Late-onset AD
MCI: Mild cognitive impairment
MEG: Magnetoencephalography
ML: Machine learning
NB: Naive Bayesian
NIA−AA: National Institute on Aging−Alzheimer’s Association
NN: Neural network
PET: Positron emission tomography
PGS: Polygenic scores
*PSEN*: Presenilins
REST: Resting-state fMRI data analysis toolkit
RF: Random forest
RL: Regression models
ROI: Regions of interest
sMCI: Stable MCI
sMRI: Structural magnetic resonance imaging
SNP: Single nucleotide polymorphism
SPM: Statistical parametric mapping
SVM: Support vector machines
VBM: Voxel-based morphometry
WM: White matter
