# Supplementary material for: A review of brain imaging biomarker genomics in Alzheimer’s disease: implementation and perspectives
Source: Transl Neurodegener. 2022 Sep 15;11:42. doi: 10.1186/s40035-022-00315-z (PMC9476275; doi:10.1186/s40035-022-00315-z)
Supplement: Supplementary file 1 — Additional file 1. Search strategy for literature. [file 40035_2022_315_MOESM1_ESM.docx]

**Supplementary Material**

**A review of brain imaging biomarker genomics in Alzheimer’s disease: implementation and perspectives**

**Supplementary methods**

*Search strategy*

Literature was searched in Google Scholar and PubMed databases. Only human studies in English language, published from January 1991 (the publication year of earliest gene cloning of *APP* mutations) to December 2021 were reviewed. The following search terms were used, where an asterisk (*) indicates a word truncation.

**PubMed**: A defined query utilizing the following keywords was used for the search in PubMed, that was, ((“genetic association”[Title/Abstract] OR “genome-wide association”[Title/Abstract] OR genotype[Title/Abstract] OR “polygenic risk”[Title/Abstract] OR “genetic risk score”[Title/Abstract]) OR ((“machine learning” OR “deep learning” OR “classification”) AND (“genetic data” OR “genotype”))) AND (AD OR Alzheimer*) AND (MRI[Title/Abstract] OR “structural MRI”[Title/Abstract] OR “functional MRI”[Title/Abstract] OR “Amyloid PET”[Title/Abstract] OR “FDG PET”[Title/Abstract] OR “Tau PET”[Title/Abstract] OR “Diffusion Tensor Imaging”[Title/Abstract]). Finally, a total of 529 relevant records were obtained from a search in PubMed.

**Google Scholar**: Various combinations with the following keywords were used in Google Scholar: (1) “Alzheimer’s disease”, “genetic association”, “polygenic risk score”, “genetic risk score”, “genotype”, “structural MRI”, “functional MRI”, “PET”, “DTI”; (2) “Alzheimer’s disease”, “genetic data”, “genotype”, “machine learning”, “deep learning”, “classification”, “structural MRI”, “functional MRI”, “FDG PET”, “Amyloid PET”, “Tau PET”, “DTI”. It should be noted that the search process was also perfected in sequence based on the search of each single imaging modal so as to reduce omissions. In total, 566 records were obtained.

Moreover, to note that, some detailed subset keyword searches have also been carried out flexibly to check and supplement additional results during paper collection, such as each single imaging modal, polygenic risk, classification with imaging and genotype data, and the like. We also identified some results by reviewing reference lists of relevant articles. Finally, the combination of the Google Scholar and PubMed databases yielded 1095 records, of which 910 records were non-duplicate.

**Abbreviations used in the text**

AD: Alzheimer’s disease; DTI: Diffusion Tensor Imaging; FDG: Fluorodeoxyglucose; MRI: Magnetic resonance imaging; PET: Positron emission tomography.
